# Bar-HRM: a reliable and fast method for species identification of ginseng (*Panax ginseng*, *Panax notoginseng*, *Talinum paniculatum* and *Phytolacca Americana*)

**DOI:** 10.7717/peerj.7660

**Published:** 2019-09-25

**Authors:** Maslin Osathanunkul, Panagiotis Madesis

**Affiliations:** 1 Department of Biology, Faculty of Science, Chiang Mai University, Chiang Mai, Thailand; 2 Center of Excellence in Bioresources for Agriculture, Industry and Medicine, Department of Biology, Faculty of Science, Chiang Mai University, Chiang Mai, Thailand; 3 Institute of Applied Biosciences, Centre for Research & Technology Hellas (CERTH), Thessaloniki, Greece

**Keywords:** Bar-HRM, Species discrimination, *Panax ginseng*, Molecular authentication, Quality control

## Abstract

**Background:**

Korean ginseng has long been famous and is one of the most well known forms of ginseng. The root of plants in the genus *Panax* is commonly recognized as ginseng. Different *Panax* species of ginseng root have been used as treatments. Although many other herbs are called ginseng, they do not contain the active compounds of ginsenosides. In Thailand, we have Thai ginseng which is of course not one of *Panax* species. Thai ginseng is the root from *Talinum paniculatum* and, due to its morphological root similarity, it is almost impossible to differentiate between them. Also, another plant species, *Phytollacca americana*, has significantly similar root morphology to real ginseng but its seeds and root are poisonous. Misunderstanding what true ginseng is compared to others could endanger lives and cause financial loss by buying inferior products.

**Methods:**

DNA barcoding combination with High Resolution Melting (called Bar-HRM) was used for species discrimination of the *Panax* ginseng and others. Five regions included ITS2, *matK*, *psbA-trnH* and *rbcL* were evaluated in the analyses.

**Results:**

The ITS2 region was found to be the most suitable primers for the analysis. The melting profile from the HRM analyses using the chosen ITS2 primers showed that Korean ginseng (*Panax ginseng*) could be discriminated from other *Penax* species. Also, other ginseng species with morphological similarity could be easily distinguished from the true ginseng. The developed Bar-HRM method poses a great potential in ginseng species discrimination and thus could be also useful in ginseng authentication.

## Introduction

The root of plants in the genus *Panax*, with the presence of ginsenosides and gintonin are typically recognized as ginseng. Thus, ginseng is actually a broad term that incorporates different species of plants belonging to the *Panax* genus e.g., Korean ginseng (*Panax ginseng*), American ginseng (*Panax quinquefolius*) and Chinese ginseng (*Panax notoginseng*). Among these, Korean ginseng is renowned for its effectiveness. However, it is very expensive and is one of the most well known ginseng (Tang & Eisenbrand, 1992; Yun, 2001; Kiefer & Pantuso, 2003). Korean ginseng (*P. ginseng*) has been studied as a way to treat Alzheimer’s disease, cancer, diabetes mellitus, heart disease, obesity, neurodegenerative disease and other conditions (Ahuja et al., 2017; Kim et al., 2018a; Kim et al., 2018b; Kim et al., 2017). Other plants from a different genus or even family were called ginseng. Although, there is some overlap in their uses, the main active compounds in ginseng from other plant genus or families differ markedly from those of *Panax* ginseng (ginsenosides). For example, the active constituents of Siberian ginseng (*Eleutherococcus senticosus*) are eleutherosides (Deyama, Nishibe & Nakazawa, 2001; Bai et al., 2011), Brazilian ginseng (*Pfaffia paniculata*) are pfaffosides (Rodrigues et al., 2013), and Indian ginseng (*Withania somnifera*) are withanolides (Mishra, Singh & Dagenais, 2000).

The root is the most medicinally valuable part of the *Panax* plant and commonly sold in dried, whole, or sliced forms whilst the leaves of *Panax* species are used on limited basis. *Panax* ginseng root can be directly consumed, or it can be included in other forms such as supplements, energy drinks, and teas. The common form of ginseng sold on worldwide market is the dried roots. It is difficult to identify plant species in these products and to differentiate the species by visual inspection of the dried root. Thus, reliable authenticating methods for medicinal plant materials become necessary. The demand for molecular approaches other than morphological identification techniques for discrimination between *Panax* species has greatly increased. Several diverse methods that do not rely on morphological characters have been successfully developed. These reported methods have been based on either DNA or protein markers (e.g.,  Choi et al., 2008; Sasaki, Komatsu & Nagumo, 2008; Lee et al., 2012; Jiang et al., 2014; Jung et al., 2014; Kim et al., 2016; Wang, Wang & Li, 2016; Yang et al., 2017). However, there are some limitations, particularly the fact that these are time-consuming. Recently, combination of two DNA-based methods, DNA barcoding and High Resolution Melting analysis, was developed, called Bar-HRM. The Bar-HRM was proven to be a reliable method for species identification and discrimination in plants (Ganopoulos, Madesis & Tsaftaris, 2012; Ganopoulos et al., 2015; Osathanunkul, Madesis & De Boer, 2015; Osathanunkul et al., 2016a; Suesatpanit et al., 2017; Osathanunkul, 2018).

In Thailand, there are some plants from other genus and family (*Talinum* and *Phytolacca*) that share a remarkable similarity in root form with *Panax* ginseng. The roots of Thai ginseng (*Talinum paniculatum*) strongly resemble those of Korean ginseng and some similar active compounds with Korean ginseng (such as steroid and terpenoid) have been reported. The root form of another plant, *Phytolacca Americana*, is the same with true ginseng, but there are reports which indicate that consuming of *P. americana* poses risks to human and mammalian health (Barnett, 1975; Lewis & Smith, 1979; Jaeckle & Freemon, 1981). Therefore, in this study, the Bar-HRM was developed for to discriminate species of true ginseng and plant species from other genus that looks very similar to it.

## Materials & Methods

### Plants samples and DNA extraction

Plant tissues including two *Phytolacca* species (*P. americana* and *P. japonica*), three *Talinum* species (*T. fruticosum*, *T. paniculata* and *T. triangulare*) and two *Panax* species (*P. ginseng* and *P. notoginseng*) were obtained from Queen Sirikit Botanic Garden (QSBG) (Table 1). The plant tissues were ground with liquid nitrogen. DNA from all samples was extracted using the Nucleospin Plant® II kit (Macherey-Nagel, Düren, Germany) according to the manufacturer’s instructions.

**Table 1:** Plant materials used in this study.

**Scientific name**	**Location/Herbarium number**	**Part**
*Panax ginseng*	Bangkok	Dry root
	Bangkok	Leaf
*Panax notoginseng*	Bangkok	Dry root
*Phytolacca americana*	QBG63283	Dry root
	Chiang Mai	Leaf
*Phytolacca japonica*	QBG33517	Dry root
*Talinum crassifolium*	QBG32481	Dry root
*Talinum fruticosum*	QBG36284	Dry root
*Talinum paniculatum*	QBG74207	Dry root
*Talinum triangulare*	QBG63253	Dry root

### Literature search

Research literature involved ginseng studies was accessed through the Web of Science Core Collection. Only publications indexed in the Science Citation Index Expanded were included in the search. Each document’s type, year of publication and corresponding author’s country were recorded. The literature search results were visualized using an open source data visualization framework called “RAWGraphs” (Mauri et al., 2017).

### Data mining for sequence analyses

The DNA sequences of plant species in genus *Phytolacca*, *Talinum* and *Panax* were searched and extracted from GenBank on National Center for Biotechnology Information (NCBI) website using the keyword “name of each genus and each chosen barcode region” (ITS, *matK*, *rbcL*, *trnL* and *trnH-psbA*). MEGA 6 program was used for sequence alignment and analysis (Tamura et al., 2013). The following characteristics were recorded: average GC content, conserved and variable site (%).

### Fragment amplification and HRM analysis

Real-time PCR amplification and DNA melting with fluorescence measurements were performed on a Rotor-Gene Q HRM system (Qiagen, Hilden, Germany). The total volume of 20 µL reaction mixture contained 20 ng genomic DNA, 10 µL of MeltDoctor™ HRM Master Mix (Applied Biosystems, Foster City, CA, USA), 0.2 µL of 10 mM forward primers and reverse primers (Table 2). Conditions are as follows; 95 °C for 5 min followed by 35 cycles of 95 °C for 30 s, 57 °C for 30 s, and 72 °C for 20 s. The temperature increased from 60 to 95 °C, at 0.1 °C/s.

**Table 2:** Sequences of primers used for fragment amplification and HRM analysis in this study.

**Primer**	**5′ → 3′**	**T**_**m**_**(°C)**	**Expected size (bp)**
HRM_ITS2F	CGCCTGCTTGGGCGTCATGGC	57	285
HRM_ITS2R	GGGCCTCGCCTGACTTGGGGCC		
HRM_matKF	CTTCTTATTTACGATTAACATCTTCT	57	170
HRM_matKR	TTTCTTTGATATCGAACATAATG		
HRM_psbA-trnHF	ATGGGGTATTGTTATTTTGTTTTG	57	115–150
HRM_psbA-trnHR	TGTATTTAATATACATATATACAATCTA		
HRM_rbcLBF	GGTACATGGACAACTGTGTGGA	57	150
HRM_rbcLBR	ACAGAACCTTCTTCAAAAAGGTCTA		
HRM_trnLF	TGGGCAATCCTGAGCCAAATC	57	120
HRM_trnLR	AACAGCTTCCATTGAGTCTCTGCACCT		

## Results

### Literature search

**Figure 1:**
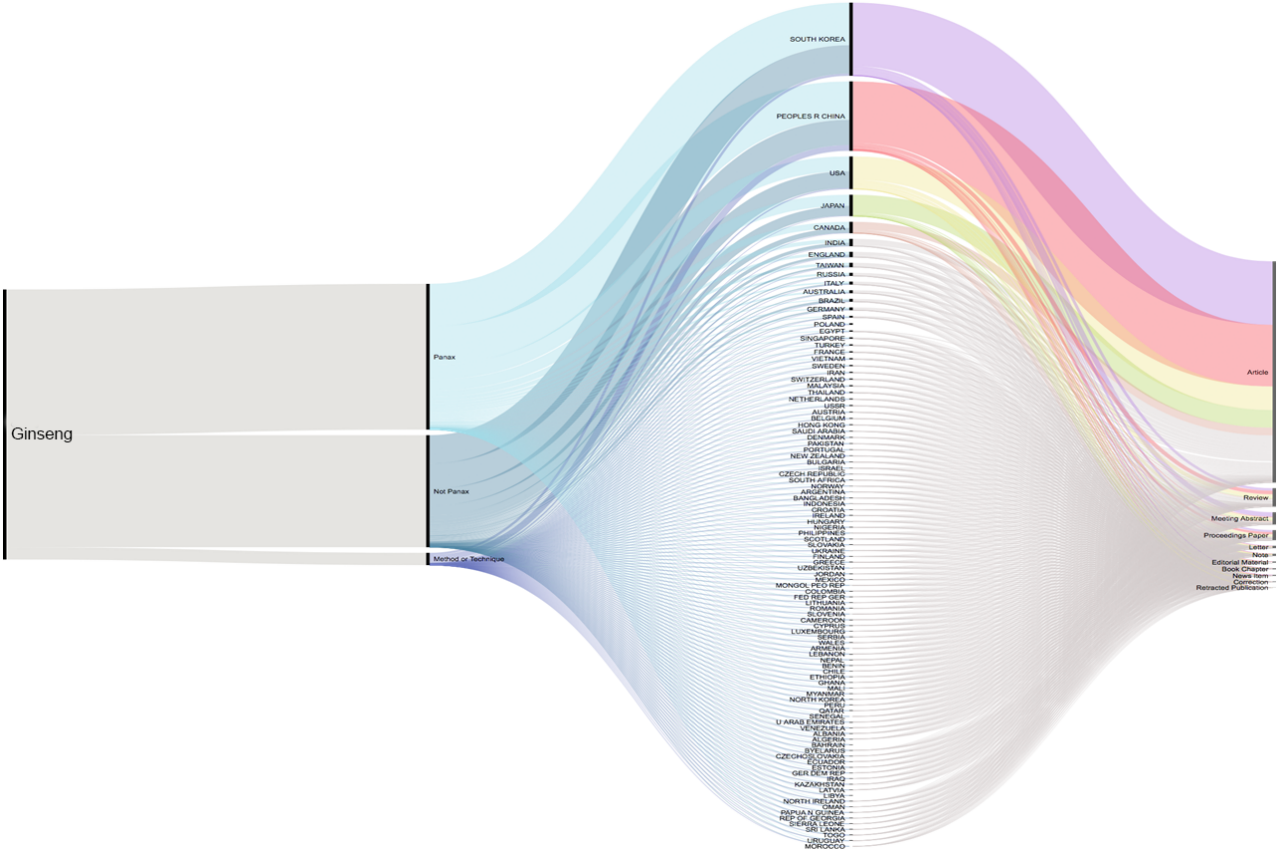
Cumulative number of ginseng studies over time (1990–2019) showing corresponding author’s country and document type.

A total of 2,724 published articles containing the word ‘ginseng’ were found when performing the literature search (July 2019), and totaling 601 other references types (reviews, proceedings, papers, meetings, abstracts, etc.) identified by our database searches (Thomson Reuters Web of Science). About 71% (1,927 articles) of the ginseng published articles were found to be about plants in *Panax* genus. Authors from South Korea have the highest contribution compared with those from other counties. Second and third to Korea were China and USA (Fig. 1). We furthered our database searches with the words ‘method’ and ‘technique’ and found that only 39 articles from 1,927 *Panax* published articles focusing on the method or technique used for species authentication/identification/discrimination.

### Data mining and *in silico* analyses

GenBank accessions were collected to assemble DNA barcode sequences of *Panax*, *Phytolacca* and *Talinum*. Data was present for most regions of the three selected genus, except for *psbA-trnH* and *trnL* of *Phytolacca* and *trnL* of *Talinum*. The total number of *Panax* sequences collected for the respective regions are as follows: 301, 196, 126, 86 and 4 species for ITS2, *matK*, *psbA-trnH*, *rbcL* and *trnL*, respectively. The total number of *Phytolacca* sequences collected for the respective regions are as follows: 6, 19, and 18 species for ITS2, *matK*, and *rbcL*, respectively. The total number of *Talinum* sequences collected for the respective regions are as follows: eight, 14, six, and eight species for ITS2, *matK*, *psbA-trnH* and *rbcL*, respectively.

Sequence length, GC content and variation within sequences lead to different T_m_ values and melting profiles which are main focus in High Resolution Melting (HRM) analysis. Therefore, all collected sequences were then analyzed using MEGA6 for average GC content (%), conserved and variable site (%). As can be seen from Table 2, the analyzed ITS2 fragment from all three plant groups was found to have a higher nucleotide variation (84.48% in *Panax* species, 8.44% in *Phytolacca* species and 21.55% in *Talinum* species) than other regions. The nucleotide variation within amplicons of *Panax* species was found to be as follows: ITS2 >*psbA-trnH* >*matK* >*rbcL* >*trnL*, *Phytolacca* species was found to be as follows: ITS2 >*matK* >*rbcL*, and *Talinum* species was found to be as follows: ITS2 >*matK* >*psbA-trnH* >*rbcL* (Table 2). It is suggested that in this study *trnL* is least suitable for ginseng species discrimination in terms of both lack of DNA data and nucleotide variation. In contrast, ITS2 poses great potential for this study. Variation in melting profiles for the different markers could also predict from an average %GC content of amplicons. The ITS2 region had the highest average %GC content in all three plant groups, with 62.4% in *Panax*, 60.8% in *Phytolacca*, and 72.2% in *Talinum* (Table 3). Based on these results, it was predicted that the ITS2 primer pair would be the best marker choice for HRM analyses with the target species.

**Table 3:** Characteristics of sequences from GenBank used in this study.

**Genus**	**Region**	**Retrieved sequence**	**Number of species**	**Analyzed fragmentlength(bp)**	**Conserved site (%)**	**Variable site (%)**	**Average GC content (%)**
*Panax*	ITS2	301	13	174	15.52	84.48	62.4
	*matK*	196	9	184	92.93	7.07	35.3
	*psbA-trnH*	126	8	352	87.78	12.22	25.9
	*rbcL*	86	8	525	91.24	8.76	43.9
	*trnL*	4	2	477	99.58	0.42	35.4
*Phytolacca*	ITS2	6	2	225	91.56	8.44	60.8
	*matK*	19	3	713	97.48	2.52	33.4
	*psbA-trnH*	0	–	–	–	–	–
	*rbcL*	18	3	476	98.95	1.05	44.4
	*trnL*	0	–	–	–	–	–
*Talinum*	ITS2	8	5	232	76.72	21.55	72.2
	*matK*	14	7	310	93.55	6.45	36.8
	*psbA-trnH*	6	3	406	96.99	3.01	34.6
	*rbcL*	8	3	481	97.71	2.29	44.3
	*trnL*	0	–	–	–	–	–

**Figure 2:**
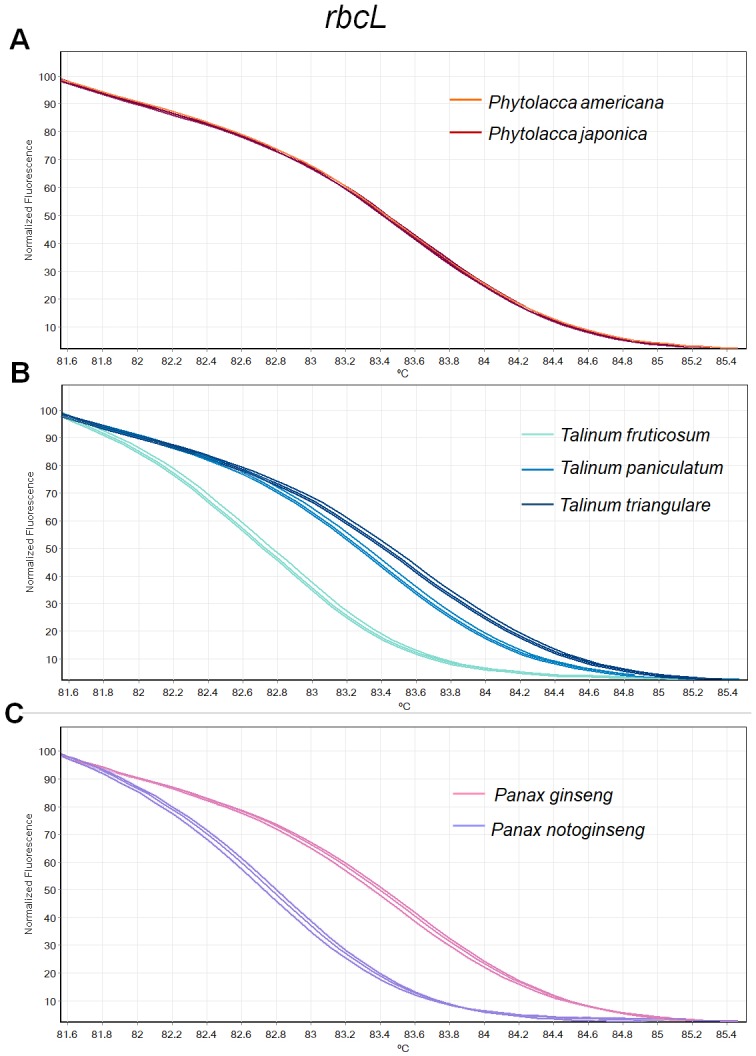
Melting curve profiles of amplicons obtained from *rbcL* primers of samples of each genus. (A) Two *Phytolacca* species (*P. americana* and *P. japonica*), (B) three *Talinum* species (*T. fruticosum*, *T. paniculata* and *T. triangulare*) and (C) two *Panax* species (*P. ginseng* and *P. notoginseng*).

### Fragment amplification for HRM analysis

**Figure 3:**
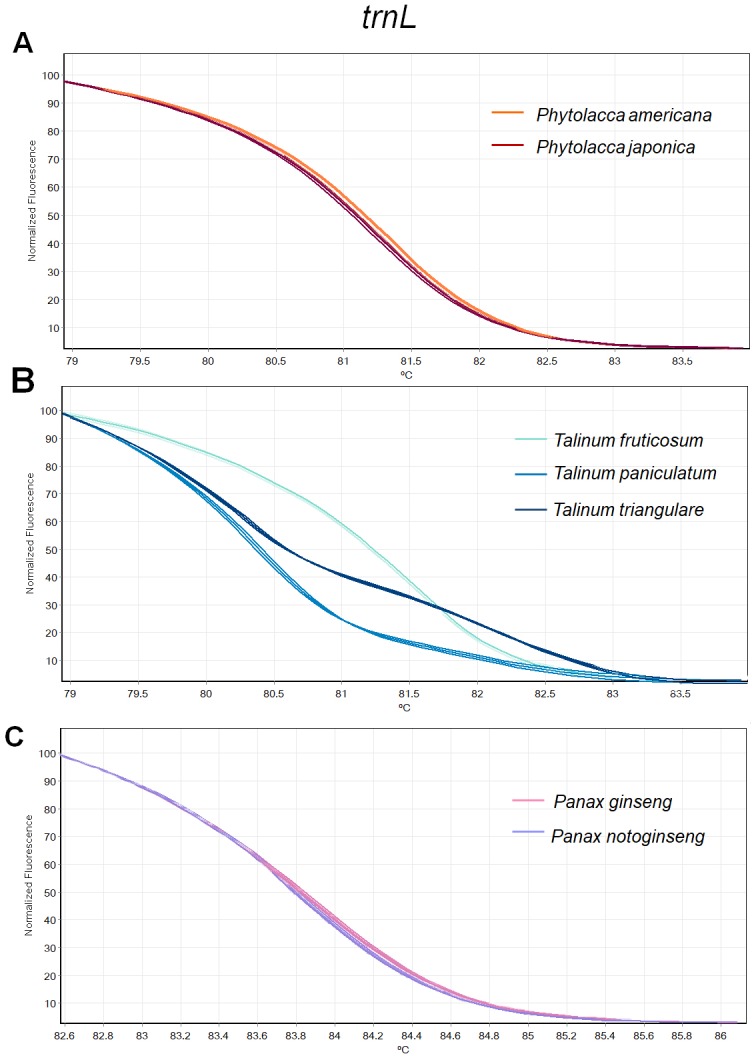
Melting curve profiles of amplicons obtained from *trnL* primers of samples of each genus. (A) Two *Phytolacca* species (*P. americana* and *P. japonica*), (B) three *Talinum* species (*T. fruticosum*, *T. paniculata* and *T. triangulare*) and (C) two *Panax* species (*P. ginseng* and *P. notoginseng*).

**Figure 4:**
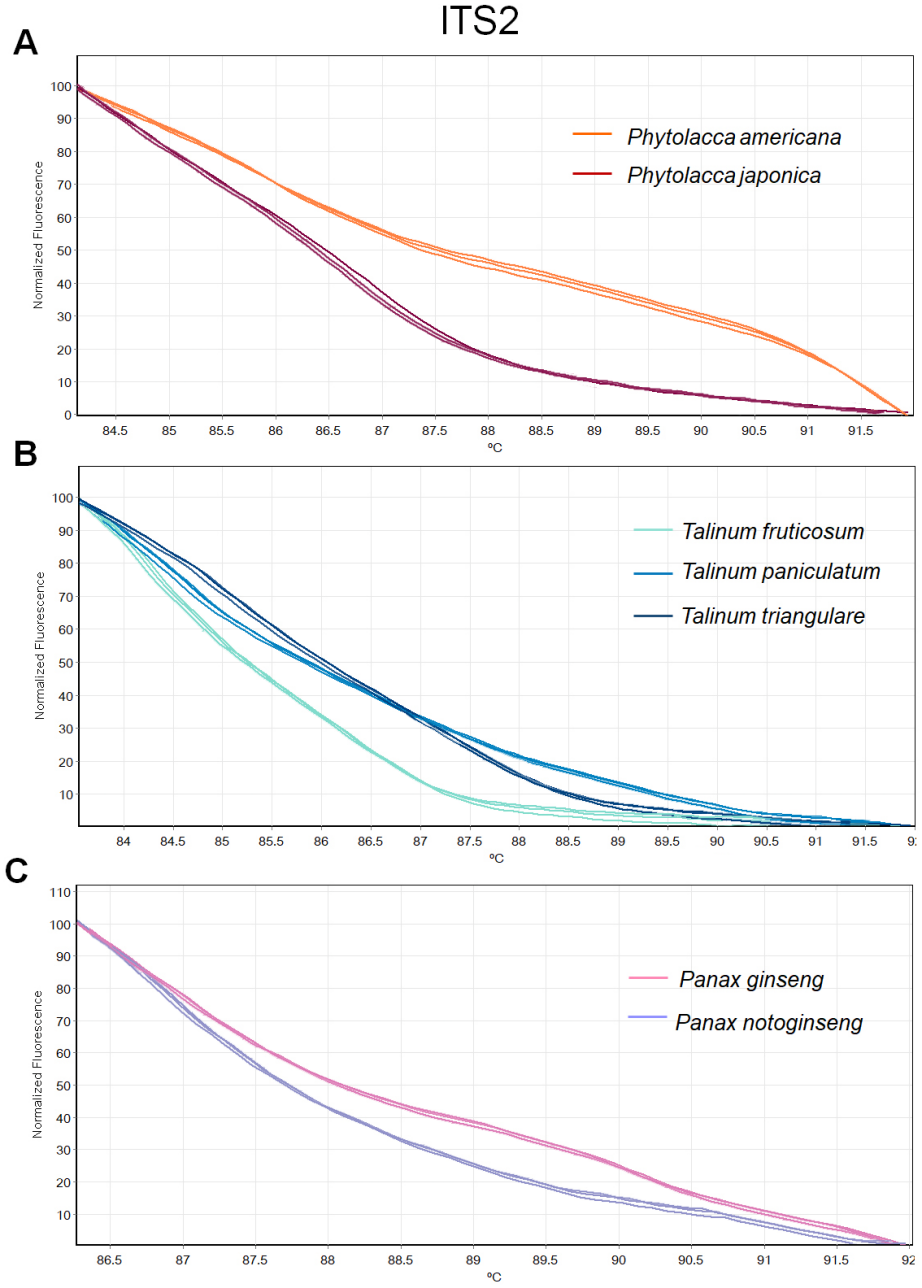
Melting curve profiles of amplicons obtained from ITS2 primers of samples of each genus. (A) Two *Phytolacca* species (*P. americana* and *P. japonica*), (B) three *Talinum* species (*T. fruticosum*, *T. paniculata* and *T. triangulare*) and (C) two *Panax* species (*P. ginseng* and *P. notoginseng*).

**Figure 5:**
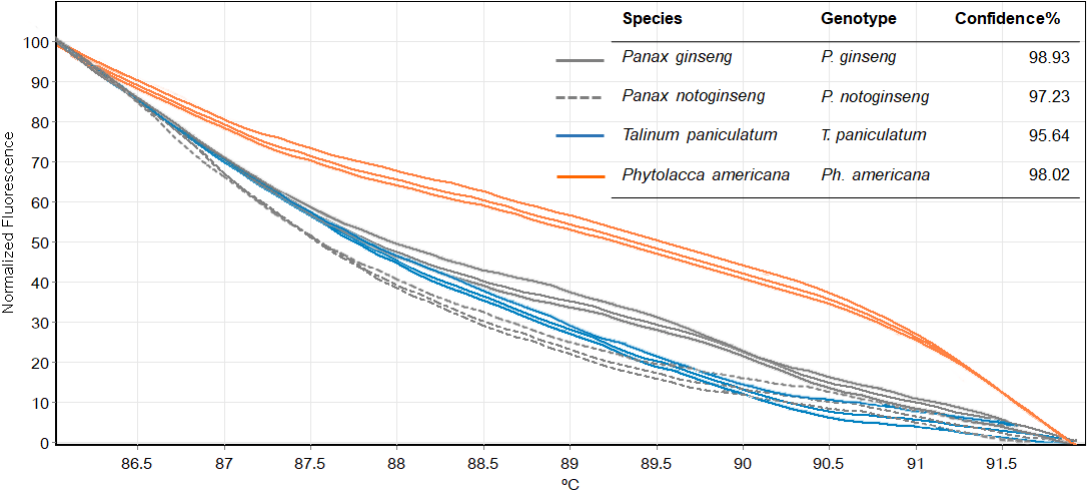
Melting curves obtained by high resolution melting analysis using ITS2 primer set of four species included *P. ginseng*, *P. notoginseng*, *P. americana* and *T. paniculatum*.

There was inconsistency in the lengths of *psbA-trnH* fragment obtained from PCR amplification due to high indel in the region. As amplicon length is one of main factors affecting HRM analysis, the *psbA-trnH* was not included here. Although *mat*K has been proposed as one of standard plant barcodes in terms of species identification, in this study HRM had a low success rate in PCR amplification with the *matK* primers. Therefore, we did not choose the *matK* for analysis here. Three primer sets including ITS2, *rbcL* and *trnL* were selected. HRM analyses was carried out in triplicate on each of the seven species including two *Phytolacca* species, three *Talinum* species and two *Panax* species to establish the melting profiles for each primer pair. The analysis is presented in T_m_ value of each species and the melting profiles of amplicons from each region are illustrated in Figs. 2–4. In this study, we also tested the hypothesis that Bar-HRM can discriminate true ginseng (*P. ginseng* and *P. notoginseng*) and other two plant species from other genus that looks very similar to the true ginseng: Thai ginseng (*T. paniculata*) and poisonous species (*P. americana*) (Fig. 5).

The HRM analysis result of *rbcL* (Figs. 2A–2C), *trnL* (Figs. 3A–3C) and ITS2 (Figs. 4A–4C) regions are shown as melting curves. The *rbcL* primers generated a unique melting curve for each *Talinum* and *Panax* species (Figs. 2B and 2C), whereas similar melting curves from two samples were observed in the *Phytolacca* species (Fig. 2A). This suggests the *rbcL* has adequate ability to discriminate the tested *Talinum* and *Panax* species but not the *Phytolacca* species. The melting curves of *P. americana* and *P. japonica* generated from *trnL* primers were nearly the same (Fig. 3A). Similarly, the shapes of the melting curves of *P. ginseng* and *P. notoginseng* were nearly identical to each other (Fig. 3C). The individual melting curves from *trnL* analysis were reproducibly obtained from each of the three different *Talinum* species (Fig. 3B). In contrast, all ITS2 amplicons from the different species of *Phytolacca*, *Talinum* and *Panax* species yielded distinctive HRM profiles (Figs. 4A–4C).

We then used only ITS2 primers in order to confirm its ability in discriminating the true ginseng (*Panax* species) from plant species that closely resemble the *Panax* ginseng in root form *T. paniculata* and *P. americana*. The melting profiles of ITS2 amplicons were shown in Fig. 5. HRM analysis based on ITS2 region can detect differences among samples and thus the two species that resemble true ginseng can be discriminated. A sequence alignment of the tested species was performed to justify the nucleotide differences between them. Within the fragment amplified by the ITS2 primers pair (285 bp in length), there were 110 variable sites found. The result here is consistent with our prediction that indicated ITS2 would be efficient in identifying the tested plant’s species. As many distributors claim false plants are part of the true ginseng family, one wonders how we can be sure about which plants the ginseng products are derived from exactly. From our results, Bar-HRM using ITS2 primers pose a great potential to authenticate the real ginseng.

## Discussion

From 2,319 published articles containing the word ‘ginseng’ in the database (Thomson Reuters Web of Science). The top three famous and popular ginseng forms are Korean ginseng (*P. ginseng*), American ginseng (*P. americana*) and Chinese ginseng (*P. notoginseng*) (Lee & Kim, 2014), this was not a surprise finding. Since the pharmacological differences within *Panax* ginseng forms have been reported, there is a call for a method or technique for authentication, identification and discrimination. However, only 2% of the *Panax* research was about the method or technique used for authentication/identification/discrimination. This is despite the fact that this research is very much in need. The roots of *Panax* ginseng have a similar appearance to each other, but they have significantly different prices, and efficacy. A rapid and reliable approach of differentiating between ginseng materials would be required for several purposes including safety and quality control (Lee & Kim, 2014). Although, several DNA-based methods have been developed such as RAPD (random amplified polymorphic DNA) (Shaw & But, 1995; Um et al., 2001), ISSR (inter-simple sequence repeat) (Bang et al., 2004; In et al., 2005), SSR (simple sequence repeat) (Kim et al., 2012), and AFLP (amplified fragment length polymorphisms) (Kim et al., 2005), these methods may produce unfavorable authentication results because of DNA degradation. Manufacturing processes could lead to DNA degradation which often prevents the recovery of PCR amplified fragments (Hajibabaei et al., 2006; Wandeler, Hoeck & Keller, 2007). In contrast, our developed Bar-HRM method are suitable for short amplified fragment analysis (∼150–300 bp). Here, we expected that our work on developing a Bar-HRM method could fill in the gap in this research field of ginseng studies.

Among the five markers which are commonly used as plant barcodes, when comparing the two most used and suggested markers for plant identification (CBO Plant Working Group, 2009), -*matK* and *rbcL-* it seems that *matK* is more suitable for the task in this study as the analysis of *rbcL* sequences showed lower number of variation than that observed in *matK*. Although, the *matK* locus is one of the most variable regions with good discriminatory power, its amplification rate is low when using standard barcoding primers which result from high substitution rates at the primer sites (CBOL Plant Working Group, 2009; Hollingsworth, 2011; Fazekas et al., 2012). ITS2 is the best marker choice for HRM analyses with our tested species. Similarly, several other DNA barcoding studies in plants have also shown the accuracy and universality of ITS (e.g., Kress et al., 2005; Fazekas et al., 2008; Chen et al., 2010; Gao et al., 2010; Li et al., 2011; Osathanunkul et al., 2018). Only three primer sets including ITS2, *rbcL* and *trnL* were selected for HRM analyses. The *matK* was excluded from this study as it has a historically low success rate (Hollingsworth, 2011). The *psbA-trnH* contains a high indel in the region that could affect HRM analysis (Osathanunkul et al., 2015; Osathanunkul et al., 2017). Although other works have found the indel polymorphism useful for discrimination of *Panax* species (Kim et al., 2013; Jung et al., 2014).

The HRM results from this study showed that the *rbcL* and *trnL* cannot be used to distinguish the *Panax* ginseng from other related species. Although, a number of works have been successfully using *rbcL* and *trnL* for identification and/or authentication of plant species (Taberlet et al., 2007; Osathanunkul, Madesis & De Boer, 2015; Osathanunkul et al., 2016a; Braukmann et al., 2017; Osathanunkul, 2018), both are not the suitable regions for the discrimination of the tested species in our study. Here, the ITS2 primers worked well for discriminating the true ginseng (*Panax* species) from plant species that closely resembled *Panax* ginseng. In contrast to our study, molecular marker analysis of ITS regions for *Panax* species has been carried by RAPD, ISSR, AFLP, and SSR techniques and found that the ITS was not a good marker for Korean ginseng identification. This is because of the low reproducibility amplifications and low polymorphism level of the ITS DNA variations (Bang et al., 2004; In et al., 2005; Kim et al., 2005). Apart from DNA markers from the chloroplast genome and internal transcribed spacer (ITS) regions, the intron site of mitochondrial cytochrome c oxidase subunit 2 (*cox2*) has been developed for authentication of Chinese ginseng (Lee et al., 2012; Wang, Wang & Li, 2016). Our previous comprehensive study indicated that the choice of marker for each study depends on the plant group in the experiment because different barcode regions were found to work well in different plant groups (Osathanunkul et al., 2016b; Osathanunkul, Osathanunkul & Madesis, 2018).

## Conclusions

Bar-HRM is quickly becoming one of the fastest developing tools currently employed for species identification and authentication. The method has proved to be efficient, rapid and reliable. It has been used to detect substitution, adulteration and the use of unreported constituents in herbal, agricultural and animal products. None of the work on Bar-HRM is targeted on authenticating real ginseng. We hypothesized that Bar-HRM poses great potential for discriminating the real ginseng from others and it is found that with the suitable choice of DNA marker, the real ginseng can be easily differentiated from closely related species or even the toxic species.

##  Supplemental Information

10.7717/peerj.7660/supp-1 File S1 Raw data (results of HRM genotyping from DNA melting)

## Additional Information and Declarations

### Data Availability

The following information was supplied regarding data availability: The raw data (DNA melting experiments) are available in File S1. All data used are available at the online database GenBank using the keyword “name of each genus and each chosen barcode region)” ITS, matK, rbcL, trnL and trnH-psbA).
