# Supplementary material for: Bar-HRM: a reliable and fast method for species identification of ginseng (Panax ginseng, Panax notoginseng, Talinum paniculatum and Phytolacca Americana)
Source: PeerJ. 2019 Sep 25;7:e7660. doi: 10.7717/peerj.7660 (PMC6765363; doi:10.7717/peerj.7660)

Raw data (results of HRM genotyping from DNA melting)

Excel Analysed Data Export
Copyright (c) 2013 QIAGEN GmbH. All Rights Reserved.

File	panax HRM_rbcL2(1).rex
Operator	
Run Id	
Notes	
Machine Serial No	814137
Channel	Gain
Green	5
Yellow	5
Orange	5
Red	5
HRM	-2.66667
Crimson	7
Channel	Threshold

HRM A.HRM (Page 1)
Cycling A.Green (Page 1)

Melt analysis of HRM A.HRM (Page 1)

No.	Color	Name	Genotype	Peak 1	Peak 2	Peak 3	Peak 4	Peak 5	Peak 6	Peak 7
1	255	QBG Phytolacca americana		60.8	61.15	61.97	62.45	63	63.53	64.23
2	4194432	QBG Phytolacca japonica		60.87	61.98	62.25	63.17	63.4	64.05	64.9
3	16711680	QBG Talinum crassifolium		60.78	61.08	61.67	62	62.38	62.95	63.55
4	13491072	QBG Talinum fruticosum		60.45	61.3	61.83	62.33	62.88	63.48	63.95
5	12615680	QBG Talinum paniculatum		60.18	60.53	61.1	61.5	62	62.53	62.95
6	16744448	QBG Talinum triangulare		60.85	61.15	61.58	61.9	62.52	63.15	63.55
7	8421376	Other		60.93	61.38	61.73	62.3	62.68	63.22	64.05
8	4227327	Panax ginseng		60.73	61.18	61.75	62.37	62.85	63.2	63.48

9	1677088	Talinum triangulare2		60.53	61.17	61.95	63.25	64	64.43	64.63
10	16711935	Talinum paniculatum2		60.28	61.38	61.9	62.43	62.82	63.6	64.28
11	197379	Talinum triangulare3		60.5	60.95	61.55	62.6	63.08	63.6	64.3
12	13158400	Talinum triangulare1		60.85	61.07	61.7	62.33	63.35	63.88	64.45
13	8404992	Phytolacca americana1		60.15	60.7	61.18	61.75	62.27	63.05	63.9
14	8510085	Phytolacca americana root dry		60.45	61.15	61.75	62.45	62.97	63.38	63.97
15	11893982	Panax ginseng root dry		60.55	60.98	61.53	61.95	62.55	62.95	63.47
16	14395776	Panax notoginseng		60.93	61.47	62.6	63.18	63.42	64.15	64.87
17	14450322	Panax notoginseng root dry		60.58	61.2	61.7	62.37	63.27	63.98	64.58
18	14515654	negative		60.45	60.7	61.22	61.77	62.43	63.15	63.83

No.	Color	Name	Genotype	Peak 8	Peak 9	Peak 10	Peak 11	Peak 12	Peak 13	Peak 14
1	255	QBG Phytolacca americana		64.75	65.1	65.58	66.13	66.55	66.98	67.45
2	4194432	QBG Phytolacca japonica		65.47	65.93	66.22	66.7	67.15	67.65	68.02
3	16711680	QBG Talinum crassifolium		63.95	64.63	65	65.6	66.08	66.35	66.65
4	13491072	QBG Talinum fruticosum		64.28	64.68	65.58	66.35	66.85	67.27	67.62
5	12615680	QBG Talinum paniculatum		63.35	63.77	64.3	64.85	65.35	65.9	66.45
6	16744448	QBG Talinum triangulare		64.37	64.88	65.3	65.88	66.53	67.4	68.12
7	8421376	Other		64.55	64.97	65.38	66.1	66.68	67.27	68
8	4227327	Panax ginseng		63.95	64.4	64.62	65.1	65.65	66.08	66.85
9	1677088	Talinum triangulare2		65.22	65.53	65.9	66.38	67.2	67.73	68.35
10	16711935	Talinum paniculatum2		64.85	65.3	65.88	66.55	66.98	67.35	67.82
11	197379	Talinum triangulare3		64.95	65.33	65.78	66.3	67.3	68.08	68.55
12	13158400	Talinum triangulare1		65	65.73	66.15	66.57	66.97	67.65	68
13	8404992	Phytolacca americana1		64.33	64.73	64.95	65.55	66.12	66.82	67.67
14	8510085	Phytolacca americana root dry		64.68	65.22	65.55	66.03	66.42	66.82	67.25
15	11893982	Panax ginseng root dry		64.23	64.85	65.25	65.9	66.45	66.97	67.35
16	14395776	Panax notoginseng		65.35	65.8	66.35	66.82	67.28	67.75	68.12
17	14450322	Panax notoginseng root dry		64.97	65.35	65.68	66.1	66.8	67.65	68.03
18	14515654	negative		64.37	64.65	65.05	65.58	66.13	66.82	67.07

No.	Color	Name	Genotype	Peak 15	Peak 16	Peak 17	Peak 18	Peak 19	Peak 20	Peak 21
1	255	QBG Phytolacca americana		68	68.95	69.5	70.17	70.62	71.08	71.73
2	4194432	QBG Phytolacca japonica		68.45	68.82	69.25	69.95	70.58	71.22	72
3	16711680	QBG Talinum crassifolium		67.03	67.35	67.75	68.17	68.75	69.3	69.75
4	13491072	QBG Talinum fruticosum		68.3	68.95	69.48	70.18	70.75	71.7	72.15
5	12615680	QBG Talinum paniculatum		66.98	67.45	67.93	68.55	69.42	69.98	70.15
6	16744448	QBG Talinum triangulare		68.65	69.18	69.87	70.55	71.1	71.95	72.45

7	8421376	Other		68.45	68.82	69.25	69.73	70.05	70.48	71.28
8	4227327	Panax ginseng		67.25	68.13	68.7	69.25	70.05	70.35	70.73
9	1677088	Talinum triangulare2		69.07	69.5	70.18	70.75	71.1	71.5	71.8
10	16711935	Talinum paniculatum2		68.48	69.2	69.85	70.35	70.97	71.9	72.25
11	197379	Talinum triangulare3		69.2	69.67	69.97	70.5	71.17	71.83	72.45
12	13158400	Talinum triangulare1		68.73	69.2	69.57	70.3	70.63	71.18	71.65
13	8404992	Phytolacca americana1		68.15	68.85	69.53	70.12	70.7	71.15	71.62
14	8510085	Phytolacca americana root dry		67.75	68.27	69	69.45	70.25	70.67	71.05
15	11893982	Panax ginseng root dry		68	68.55	69.4	70.08	71.15	71.62	71.85
16	14395776	Panax notoginseng		68.67	69.25	69.7	69.9	70.53	71.1	72
17	14450322	Panax notoginseng root dry		68.35	68.75	69.38	69.9	70.35	71.35	71.72
18	14515654	negative		67.62	68.27	68.8	68.95	69.65	70.27	70.72

No.	Color	Name	Genotype	Peak 22	Peak 23	Peak 24	Peak 25	Peak 26	Peak 27	Peak 28
1	255	QBG Phytolacca americana		72.6	73.3	73.83	74.5	75.35	76.55	77.88
2	4194432	QBG Phytolacca japonica		72.47	73.17	73.97	74.85	75.25	75.85	76.33
3	16711680	QBG Talinum crassifolium		70.25	70.73	71.45	72.03	72.4	72.93	73.65
4	13491072	QBG Talinum fruticosum		72.77	73.08	73.67	74.15	74.5	75.3	76.2
5	12615680	QBG Talinum paniculatum		70.45	71	71.63	72.32	72.8	73.2	73.8
6	16744448	QBG Talinum triangulare		72.65	73.3	73.92	74.42	75.2	75.67	76.4
7	8421376	Other		71.9	72.45	72.95	73.52	73.97	74.25	74.6
8	4227327	Panax ginseng		71.18	71.72	72.15	72.53	72.93	73.25	73.65
9	1677088	Talinum triangulare2		72.3	72.88	73.6	74.2	75.1	75.72	76.17
10	16711935	Talinum paniculatum2		72.62	73.25	73.88	74.6	75.33	75.75	76.3
11	197379	Talinum triangulare3		73.37	73.7	74.55	74.98	75.3	75.68	76.25
12	13158400	Talinum triangulare1		72.15	72.43	72.98	73.25	73.55	73.83	74.35
13	8404992	Phytolacca americana1		72.05	72.9	73.62	73.97	74.75	75.27	75.87
14	8510085	Phytolacca americana root dry		71.63	72.6	73.08	73.7	74.23	74.57	75.2
15	11893982	Panax ginseng root dry		72.27	72.7	73.28	73.93	74.25	74.93	75.7
16	14395776	Panax notoginseng		72.7	73.4	73.93	74.72	75.05	75.4	76.42
17	14450322	Panax notoginseng root dry		72.12	72.45	72.95	73.6	73.85	74.35	74.77
18	14515654	negative		71.65	73	73.62	74.45	75.45	75.88	76.92

No.	Color	Name	Genotype	Peak 29	Peak 30	Peak 31	Peak 32	Peak 33	Peak 34	Peak 35
1	255	QBG Phytolacca americana		78.35	78.83	79.27	79.67	81.28	81.88	83.63
2	4194432	QBG Phytolacca japonica		76.68	77.08	77.5	78.3	78.9	79.47	81.23
3	16711680	QBG Talinum crassifolium		73.98	74.55	74.95	75.37	75.92	76.45	76.88
4	13491072	QBG Talinum fruticosum		76.92	77.4	78.42	78.7	79.3	80.55	82.73

5	12615680	QBG Talinum paniculatum		74.3	75.15	75.73	76.35	76.75	77.2	77.55
6	16744448	QBG Talinum triangulare		77.1	77.58	77.97	78.35	79.3	79.95	80.47
7	8421376	Other		75.02	75.35	75.9	76.65	77.45	77.9	78.15
8	4227327	Panax ginseng		74.02	74.38	74.95	75.57	75.95	76.65	76.87
9	1677088	Talinum triangulare2		76.95	77.35	77.93	78.7	79.25	79.73	80.07
10	16711935	Talinum paniculatum2		77.15	77.92	78.5	79.1	80.88	81.27	81.93
11	197379	Talinum triangulare3		77.1	77.98	78.45	79.4	80	80.45	80.93
12	13158400	Talinum triangulare1		75	75.5	75.95	76.65	77.05	77.43	78.08
13	8404992	Phytolacca americana1		76.43	76.92	77.38	78.03	78.42	78.72	79.25
14	8510085	Phytolacca americana root dry		75.72	76.18	76.6	77.25	77.55	78	78.3
15	11893982	Panax ginseng root dry		76.3	76.77	77.2	77.6	77.93	78.4	79.15
16	14395776	Panax notoginseng		76.95	77.55	78.23	78.85	79.38	79.88	80.38
17	14450322	Panax notoginseng root dry		75.4	75.9	76.27	76.87	77.42	77.78	78.47
18	14515654	negative		77.38	77.82	78.33	78.65	79.1	79.72	80.05

No.	Color	Name	Genotype	Peak 36	Peak 37	Peak 38	Peak 39	Peak 40	Peak 41	Peak 42
1	255	QBG Phytolacca americana		86.2	86.78	87.23	87.63	88.1	88.8	89.1
2	4194432	QBG Phytolacca japonica		81.75	82.22	83.52	85.9	86.43	86.92	87.58
3	16711680	QBG Talinum crassifolium		77.4	78.17	78.6	79.25	79.65	80.25	81.48
4	13491072	QBG Talinum fruticosum		83	84.8	85.23	86.63	87.17	87.73	
5	12615680	QBG Talinum paniculatum		77.95	78.87	79.33	79.95	81.28	81.8	83.5
6	16744448	QBG Talinum triangulare		81.3	81.95	83.5	85.97	86.9		
7	8421376	Other		78.45	78.9	80.25	80.78	81.03	83.4	85.52
8	4227327	Panax ginseng		77.35	77.75	78.18	79.18	81.28	81.75	83.6
9	1677088	Talinum triangulare2		81.2	83.4					
10	16711935	Talinum paniculatum2		83.47	85.3	85.83	86.23	86.75	87.2	87.8
11	197379	Talinum triangulare3		81.25	81.98	83.57	85.95			
12	13158400	Talinum triangulare1		78.97	80.25	81.1	83.55	85.97	87	
13	8404992	Phytolacca americana1		81.25	81.85	83.63	86.28	86.65	87.28	87.77
14	8510085	Phytolacca americana root dry		78.7	79.13	80.05	80.7	81.13	81.5	82
15	11893982	Panax ginseng root dry		79.92	80.45	81.27	81.83	83.58	85.78	86.18
16	14395776	Panax notoginseng		82.73	85.48	86.03	86.45	86.95	87.5	87.9
17	14450322	Panax notoginseng root dry		79.03	79.65	80.25	82.77			
18	14515654	negative		81.15	81.62	82.6	83.32	83.9	84.93	85.22

No.	Color	Name	Genotype	Peak 43	Peak 44	Peak 45	Peak 46	Peak 47	Peak 48	Peak 49
1	255	QBG Phytolacca americana		89.7						
2	4194432	QBG Phytolacca japonica		88.18	89.27	89.58				

3	16711680	QBG Talinum crassifolium		81.83	82.47	84.08	86.68	88.47		
4	13491072	QBG Talinum fruticosum								
5	12615680	QBG Talinum paniculatum		85.75	86.2	86.82	87.28	87.72	88.23	88.6
6	16744448	QBG Talinum triangulare								
7	8421376	Other		85.95	86.37	87.02	88.15	89.52		
8	4227327	Panax ginseng		86.3	86.75	88.75				
9	1677088	Talinum triangulare2								
10	16711935	Talinum paniculatum2		88.5	89.05	89.47	89.77			
11	197379	Talinum triangulare3								
12	13158400	Talinum triangulare1								
13	8404992	Phytolacca americana1		88.45	88.67	89.17	89.82			
14	8510085	Phytolacca americana root dry		83.57	86.23	86.72	87.55			
15	11893982	Panax ginseng root dry		86.87	87.45	87.95	89.12	89.35		
16	14395776	Panax notoginseng		88.27	89	89.52				
17	14450322	Panax notoginseng root dry								
18	14515654	negative		85.7	86.25	86.65	87.28	87.65	88.55	

No.	Color	Name	Genotype	Peak 50	Peak 51
1	255	QBG Phytolacca americana			
2	4194432	QBG Phytolacca japonica			
3	16711680	QBG Talinum crassifolium			
4	13491072	QBG Talinum fruticosum			
5	12615680	QBG Talinum paniculatum		89.02	89.42
6	16744448	QBG Talinum triangulare			
7	8421376	Other			
8	4227327	Panax ginseng			
9	1677088	Talinum triangulare2			
10	16711935	Talinum paniculatum2			
11	197379	Talinum triangulare3			
12	13158400	Talinum triangulare1			
13	8404992	Phytolacca americana1			
14	8510085	Phytolacca americana root dry			
15	11893982	Panax ginseng root dry			
16	14395776	Panax notoginseng			
17	14450322	Panax notoginseng root dry			
18	14515654	negative			

rbcL(1)

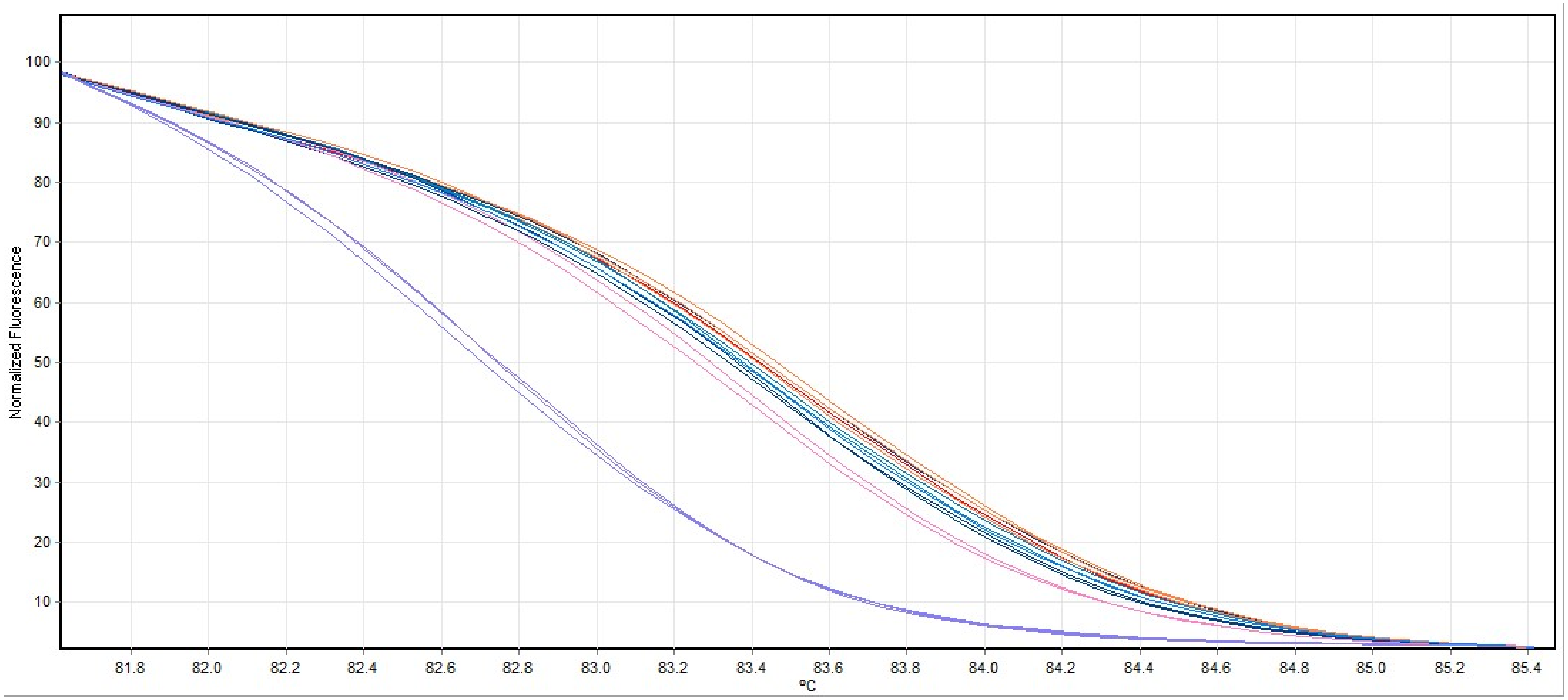

Excel Analysed Data Export
Copyright (c) 2013 QIAGEN GmbH. All Rights Reserved.

File	panax HRM_rbcL2(2).rex

Operator
Run Id
Notes
Machine Serial No 814137

Channel	Gain
Green	5

Yellow	5
Orange	5
Red	5
HRM	-2.66667
Crimson	7

Channel	Threshold
HRM A.HRM (Page	0.13493
Cycling A.Green (Page 1)	
Cycling A.HRM (Page 1)	

Melt analysis of HRM A.HRM (Page 1)

No.	Color	Name	Genotype	Peak 1	Peak 2	Peak 3	Peak 4	Peak 5	Peak 6
1	255	QBG Phytolacca americana		60.85	61.21	62.1	62.57	62.96	63.71
2	4194432	QBG Phytolacca japonica		60.6	61.79	62.38	63.22	63.55	64.24
3	16711680	QBG Talinum crassifolium		60.91	61.1	61.55	62.11	62.41	63.1
4	13491072	QBG Talinum fruticosum		60.56	61.46	61.62	62.3	62.9	63.33
5	12615680	QBG Talinum paniculatum		60.3	60.72	61.15	61.65	62.14	62.4
6	16744448	QBG Talinum triangulare		60.77	61.2	61.62	61.89	62.25	63.07
7	8421376	Other		60.82	61.22	61.71	62.33	62.7	63.43
8	4227327	Panax ginseng		60.61	61.29	61.88	62.5	62.91	63.21
9	1677088	Talinum triangulare2		60.7	61.28	61.67	63.57	64.1	64.37
10	16711935	Talinum paniculatum2		60.32	61.46	61.86	62.18	62.98	63.52
11	197379	Talinum triangulare3		60.69	60.98	61.48	62.67	63.2	63.9
12	13158400	Talinum triangulare1		60.851	61.11	61.76	62.2	63.43	63.67
13	8404992	Phytolacca americana1		60.34	60.81	61.4	61.77	62.12	63.17
14	8510085	Phytolacca americana root dry		60.27	61.08	61.39	62.3	62.9	63.44
15	11893982	Panax ginseng root dry		60.59	60.79	61.61	61.99	62.34	62.87
16	14395776	Panax notoginseng		60.87	61.25	62.2	63.42	63.7	64.2
17	14450322	Panax notoginseng root dry		60.65	61.34	61.65	62.18	63.76	64.65
18	14515654	negative							

No.	Color	Name	Genotype	Peak 8	Peak 9	Peak 10	Peak 11	Peak 12	Peak 13
1	255	QBG Phytolacca americana		64.75	65.1	65.58	66.13	66.55	66.98
2	4194432	QBG Phytolacca japonica		65.47	65.93	66.22	66.7	67.15	67.65

3	16711680	QBG Talinum crassifolium		63.95	64.63	65	65.6	66.08	66.35
4	13491072	QBG Talinum fruticosum		64.28	64.68	65.58	66.35	66.85	67.27
5	12615680	QBG Talinum paniculatum		63.35	64.3	65.35	67.45	79.95	81.28
6	16744448	QBG Talinum triangulare		64.37	64.88	65.3	65.88	66.53	67.4
7	8421376	Other		64.55	64.97	65.38	66.1	66.68	67.27
8	4227327	Panax ginseng		63.95	64.4	64.62	65.1	65.65	66.08
9	1677088	Talinum triangulare2		65.22	65.53	65.9	66.38	67.2	67.73
10	16711935	Talinum paniculatum2		64.85	65.3	65.88	66.55	66.98	67.35
11	197379	Talinum triangulare3		64.95	65.33	65.78	66.3	67.3	68.08
12	13158400	Talinum triangulare1		65	65.73	66.15	66.57	66.97	67.65
13	8404992	Phytolacca americana1		64.33	64.73	64.95	65.55	66.12	66.82
14	8510085	Phytolacca americana root dry		64.68	65.22	65.55	66.03	66.42	66.82
15	11893982	Panax ginseng root dry		64.23	64.85	65.25	65.9	66.45	66.97
16	14395776	Panax notoginseng		65.35	65.8	66.35	66.82	67.28	67.75
17	14450322	Panax notoginseng root dry							
18	14515654	negative							

No.	Color	Name	Genotype	Peak 15	Peak 16	Peak 17	Peak 18	Peak 19	Peak 20
1	255	QBG Phytolacca americana		68	68.95	69.5	70.17	71.08	71.73
2	4194432	QBG Phytolacca japonica		68.45	68.82	69.25	69.95	70.58	71.22
3	16711680	QBG Talinum crassifolium		67.03	67.35	67.75	68.17	68.75	69.3
4	13491072	QBG Talinum fruticosum		68.3	68.95	69.48	76.2	78.7	79.3
5	12615680	QBG Talinum paniculatum		83.5					
6	16744448	QBG Talinum triangulare		68.65	69.18	69.87	70.55	79.3	79.95
7	8421376	Other		68.45	68.82	69.25	69.73	70.05	70.48
8	4227327	Panax ginseng		67.25	68.13	68.7	69.25	70.05	70.35
9	1677088	Talinum triangulare2		69.5	79.73	80.07	81.2	83.4	
10	16711935	Talinum paniculatum2		68.48	69.2	69.85	70.35	70.97	71.9
11	197379	Talinum triangulare3		69.2	69.67	69.97	70.5	71.17	72.45
12	13158400	Talinum triangulare1		68.73	69.2	70.3	80.25	81.1	83.55
13	8404992	Phytolacca americana1		68.15	68.85	69.53	70.12	70.7	71.62
14	8510085	Phytolacca americana root dry		67.75	68.27	69	69.45	70.25	70.67
15	11893982	Panax ginseng root dry		68	68.55	69.4	70.08	71.15	72.27
16	14395776	Panax notoginseng		68.67	69.25	69.7	69.9	70.53	72.7
17	14450322	Panax notoginseng root dry							
18	14515654	negative							

No.	Color	Name	Genotype	Peak 22	Peak 23	Peak 24	Peak 25	Peak 26	Peak 27

1	255	QBG Phytolacca americana		73.3	73.83	76.55	79.27	79.67	81.28
2	4194432	QBG Phytolacca japonica		72.47	73.17	73.97	77.5	78.3	79.47
3	16711680	QBG Talinum crassifolium		70.25	70.73	71.45	72.03	72.4	72.93
4	13491072	QBG Talinum fruticosum		82.73	83				
5	12615680	QBG Talinum paniculatum							
6	16744448	QBG Talinum triangulare		81.3	81.95	83.5			
7	8421376	Other		72.45	80.25	80.78	81.03	83.4	
8	4227327	Panax ginseng		71.18	71.72	75.95	78.18	79.18	81.28
9	1677088	Talinum triangulare2							
10	16711935	Talinum paniculatum2		73.25	74.6	75.33	75.75	77.92	78.5
11	197379	Talinum triangulare3		74.55	74.98	75.3	79.4	80	80.45
12	13158400	Talinum triangulare1							
13	8404992	Phytolacca americana1		72.9	73.62	73.97	75.87	76.92	78.03
14	8510085	Phytolacca americana root dry		71.63	73.08	76.18	79.13	80.05	80.7
15	11893982	Panax ginseng root dry		79.15	79.92	80.45	81.27	81.83	83.58
16	14395776	Panax notoginseng		73.93	75.4	78.85	79.38	80.38	82.73
17	14450322	Panax notoginseng root dry							
18	14515654	negative							

No.	Color	Name	Genotype	Peak 29	Peak 30	Peak 31	Peak 32	Peak 33	Peak 34
1	255	QBG Phytolacca americana		83.63					
2	4194432	QBG Phytolacca japonica		81.75	82.22	83.52			
3	16711680	QBG Talinum crassifolium		73.98	77.4	79.25	79.65	80.25	81.48
4	13491072	QBG Talinum fruticosum							
5	12615680	QBG Talinum paniculatum							
6	16744448	QBG Talinum triangulare							
7	8421376	Other							
8	4227327	Panax ginseng		83.6					
9	1677088	Talinum triangulare2							
10	16711935	Talinum paniculatum2		80.88	81.27	81.93	83.47		
11	197379	Talinum triangulare3		81.25	81.98	83.57			
12	13158400	Talinum triangulare1							
13	8404992	Phytolacca americana1		81.25	81.85	83.63			
14	8510085	Phytolacca americana root dry		81.5	82	83.57			
15	11893982	Panax ginseng root dry							
16	14395776	Panax notoginseng							
17	14450322	Panax notoginseng root dry							

18 14515654 negative

No.	Color	Name	Genotype	Peak 36	Peak 37
1	255	QBG Phytolacca americana			
2	4194432	QBG Phytolacca japonica			
3	16711680	QBG Talinum crassifolium		82.47	84.08
4	13491072	QBG Talinum fruticosum			
5	12615680	QBG Talinum paniculatum			
6	16744448	QBG Talinum triangulare			
7	8421376	Other			
8	4227327	Panax ginseng			
9	1677088	Talinum triangulare2			
10	16711935	Talinum paniculatum2			
11	197379	Talinum triangulare3			
12	13158400	Talinum triangulare1			
13	8404992	Phytolacca americana1			
14	8510085	Phytolacca americana root dry			
15	11893982	Panax ginseng root dry			
16	14395776	Panax notoginseng			
17	14450322	Panax notoginseng root dry			
18	14515654	negative			

rbcL(2)

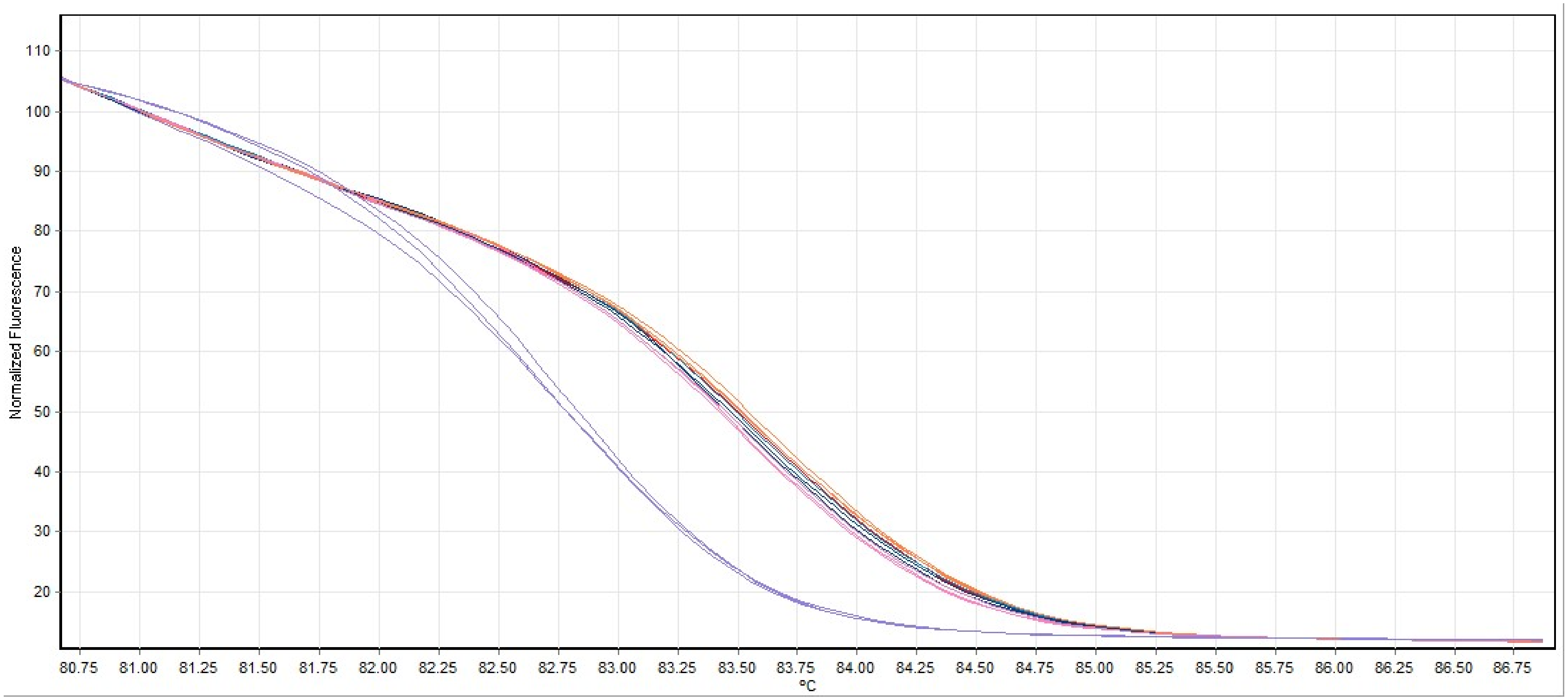

Excel Analysed Data Export

Copyright (c) 2013 QIAGEN GmbH. All Rights Reserved.

File	panax HRM_rbcL2(3).rex
Operator	
Run Id	
Notes	
Machine Serial No	814137

Channel	Gain
Green	5
Yellow	5

Orange	5
Red	5
HRM	-2.66667
Crimson	7

Channel	Threshold
HRM A.HRM (Page	0.04639
Cycling A.Green (Page 1)	
Cycling A.HRM (Page 1)	

Melt analysis of HRM A.HRM (Page 1)

No.	Color	Name	Genotype	Peak 1	Peak 2	Peak 3	Peak 4	Peak 5	Peak 6	Peak 7
1	255	QBG Phytolacca americana		60.77	61.23	61.84	62.67	63.1	63.67	64.08
2	4194432	QBG Phytolacca japonica		60.92	61.79	62.3	63.09	63.22	64.12	64.83
3	16711680	QBG Talinum crassifolium		60.69	61.2	61.77	62.15	62.48	62.88	63.41
4	13491072	QBG Talinum fruticosum		60.6	61.52	61.56	62.37	62.76	63.21	63.76
5	12615680	QBG Talinum paniculatum		60.24	60.61	61.24	61.62	62.04	62.47	62.84
6	16744448	QBG Talinum triangulare		60.71	61.32	61.61	61.84	62.48	63.3	63.61
7	8421376	Other		60.83	61.44	61.92	62.47	62.77	63.51	64.18
8	4227327	Panax ginseng		60.68	61.3	61.67	62.14	62.91	63.17	63.31
9	1677088	Talinum triangulare1		60.64	61.23	62.1	63.19	64.07	64.38	64.7
10	16711935	Talinum paniculatum1		60.21	61.34	61.83	62.12	62.94	63.51	64.16
11	197379	Talinum triangulare3		60.63	60.89	61.7	62.74	63.16	63.43	64.45
12	13158400	Talinum triangulare2		60.78	61.22	61.88	62.21	63.2	63.77	64.36
13	8404992	Phytolacca americana1		60.51	60.87	61.23	61.88	62.44	63.16	63.84
14	8510085	Phytolacca americana1		60.48	61.42	61.56	62.36	62.81	63.24	64.1
15	11893982	Panax ginseng root dry		60.59	60.86	61.64	62.1	62.62	62.87	63.23
16	14395776	Panax notoginseng		60.9	61.21	62.71	63.2	63.71	64.08	64.66
17	14450322	Panax notoginseng root dry		60.82	61.33	61.89	62.44	63.6	63.95	64.71
18	14515654	dH2O								

No.	Color	Name	Genotype	Peak 8	Peak 9	Peak 10	Peak 11	Peak 12	Peak 13	Peak 14
1	255	QBG Phytolacca americana		64.61	65.27	65.81	66.19	66.63	67	67.57
2	4194432	QBG Phytolacca japonica		65.32	65.81	66.09	66.68	67.07	67.49	68.1
3	16711680	QBG Talinum crassifolium		63.8	64.57	65.11	65.72	66.29	66.34	66.82

4	13491072	QBG Talinum fruticosum		64.37	64.71	65.3	66.41	66.9	67	67.51
5	12615680	QBG Talinum paniculatum		63.45	64.12	64.37	64.97	65.31	65.62	66.41
6	16744448	QBG Talinum triangulare		64.32	64.52	64.85	64.97	65.12	65.69	66.04
7	8421376	Other		64.5	65.13	65.57	66.25	66.62	67.1	67.65
8	4227327	Panax ginseng		63.7	64.25	64.71	65.02	65.68	66.15	66.62
9	1677088	Talinum triangulare1		65.13	65.57	66.25	66.62	67.1	67.65	68.33
10	16711935	Talinum paniculatum1		64.65	65.25	65.6	66.2	66.6	67.15	67.67
11	197379	Talinum triangulare3		65.47	66.2	66.75	67.2	67.65	68.35	68.92
12	13158400	Talinum triangulare2		65.37	65.85	66.33	66.68	67.43	68.22	68.76
13	8404992	Phytolacca americana1		64.43	64.71	64.96	65.61	66.19	66.37	66.87
14	8510085	Phytolacca americana1		64.45	64.88	65.28	65.58	66.3	66.55	66.77
15	11893982	Panax ginseng root dry		64.55	65.38	66.2	66.81	67.2	67.65	68.35
16	14395776	Panax notoginseng		64.95	65.35	65.73	66.55	66.89	67.1	67.55
17	14450322	Panax notoginseng root dry		64.65	65.05	65.58	66.13	66.82	67.07	67.83
18	14515654	dH2O								

No.	Color	Name	Genotype	Peak 15	Peak 16	Peak 17	Peak 18	Peak 19	Peak 20	Peak 21
1	255	QBG Phytolacca americana		68.11	68.89	69.47	70.23	70.71	71.2	71.87
2	4194432	QBG Phytolacca japonica		68.56	68.97	69.19	69.87	70.42	71.19	71.78
3	16711680	QBG Talinum crassifolium		67.3	67.87	68.02	68.36	68.89	69.15	69.7
4	13491072	QBG Talinum fruticosum		68.19	68.92	69.47	70.1	70.62	71.54	72.07
5	12615680	QBG Talinum paniculatum		67.25	67.47	68.81	69.28	69.58	70.04	70.29
6	16744448	QBG Talinum triangulare		66.87	67.21	68.36	69.64	71.23	71.85	72.13
7	8421376	Other		68.33	68.85	69.35	69.84	70.21	70.63	71.1
8	4227327	Panax ginseng		67.3	67.82	68.55	69.09	69.98	70.12	70.68
9	1677088	Talinum triangulare1		68.85	69.35	70.1	70.66	71.09	71.43	71.96
10	16711935	Talinum paniculatum1		68.33	69.17	69.63	70.38	71.1	71.67	72.38
11	197379	Talinum triangulare3		69.5	69.88	69.97	70.24	71.02	71.83	72.22
12	13158400	Talinum triangulare2		69.35	69.81	69.98	70.17	70.7	71.65	72.1
13	8404992	Phytolacca americana1		67.4	68.42	69.27	70.1	70.38	71.08	71.78
14	8510085	Phytolacca americana1		67.15	68.31	69.1	69.67	70.25	70.77	71.17
15	11893982	Panax ginseng root dry		68.92	69.3	69.75	70.24	71.32	71.74	72.04
16	14395776	Panax notoginseng		68.24	68.84	69.32	69.74	70.6	71.18	71.96
17	14450322	Panax notoginseng root dry		68.19	68.81	69.27	70.2	70.48	71.34	71.92
18	14515654	dH2O								

No.	Color	Name	Genotype	Peak 22	Peak 23	Peak 24	Peak 25	Peak 26	Peak 27	Peak 28
1	255	QBG Phytolacca americana		72.85	73.47	73.9	74.79	75.16	75.63	76.18

2	4194432	QBG Phytolacca japonica		72.28	72.87	73.41	73.96	74.71	75.2	75.74
3	16711680	QBG Talinum crassifolium		70.37	70.95	71.4	72.3	72.75	73.18	73.68
4	13491072	QBG Talinum fruticosum		72.69	73.11	73.62	74.27	74.69	75.47	76.41
5	12615680	QBG Talinum paniculatum		70.68	71.12	71.87	72.31	72.94	73.36	73.92
6	16744448	QBG Talinum triangulare		72.57	73.18	73.82	74.33	75.01	75.72	76.14
7	8421376	Other		71.87	72.34	72.89	73.41	73.83	74.1	74.77
8	4227327	Panax ginseng		71.2	71.84	72.08	72.41	72.74	73.18	73.49
9	1677088	Talinum triangulare1		72.18	72.97	73.6	74.2	75.1	75.72	76.17
10	16711935	Talinum paniculatum1		72.8	73.19	73.88	74.6	75.33	75.75	76.3
11	197379	Talinum triangulare3		72.64	73.42	74.55	74.98	75.3	75.68	76.25
12	13158400	Talinum triangulare2		72.8	73.25	72.98	73.25	73.55	73.83	74.35
13	8404992	Phytolacca americana1		72.24	73.04	73.71	73.89	74.62	75.08	75.87
14	8510085	Phytolacca americana1		71.69	72.47	73.1	73.61	74.57	75.17	75.41
15	11893982	Panax ginseng root dry		72.38	72.91	73.14	73.87	74.19	74.86	75.57
16	14395776	Panax notoginseng		72.64	73.28	73.85	74.62	75.17	75.56	76.2
17	14450322	Panax notoginseng root dry		72.44	72.87	73.2	73.89	74.14	74.57	74.98
18	14515654	dH2O								

No.	Color	Name	Genotype	Peak 29	Peak 30	Peak 31	Peak 32	Peak 33	Peak 34	Peak 35
1	255	QBG Phytolacca americana		76.7	77.44	78.65	79.61	81.07	81.67	83.52
2	4194432	QBG Phytolacca japonica		76.27	77.15	77.9	78.25	78.87	79.31	81.1
3	16711680	QBG Talinum crassifolium		74.2	74.71	75.19	75.57	75.89	76.21	76.92
4	13491072	QBG Talinum fruticosum		76.88	77.34	78.21	78.63	79.12	80.24	82.67
5	12615680	QBG Talinum paniculatum		74.27	75.28	75.81	76.22	76.84	77.38	77.61
6	16744448	QBG Talinum triangulare		77.09	77.41	77.82	78.28	79.15	79.95	80.47
7	8421376	Other		75.18	75.52	75.92	76.42	77.25	77.87	78.4
8	4227327	Panax ginseng		73.98	74.25	74.87	75.41	75.79	76.52	76.79
9	1677088	Talinum triangulare1		76.95	77.35	77.93	78.7	79.25	79.73	80.07
10	16711935	Talinum paniculatum1		77.15	77.92	78.5	79.1	80.88	81.27	81.93
11	197379	Talinum triangulare3		77.1	77.98	78.45	79.4	80	80.45	80.93
12	13158400	Talinum triangulare2		75	75.5	75.95	76.65	77.05	77.43	78.08
13	8404992	Phytolacca americana1		76.43	76.92	77.38	78.03	78.42	78.72	79.25
14	8510085	Phytolacca americana1		75.94	76.71	77.09	77.93	78.16	78.92	79.1
15	11893982	Panax ginseng root dry		76.18	76.61	77.13	77.48	77.82	78.25	79.08
16	14395776	Panax notoginseng		76.87	77.41	78.09	78.83	79.16	79.88	80.38
17	14450322	Panax notoginseng root dry		75.39	75.78	76.27	76.87	77.42	77.78	78.47
18	14515654	dH2O								

No.	Color	Name	Genotype	Peak 36	Peak 37	Peak 38	Peak 39	Peak 40	Peak 41	Peak 42
1	255	QBG Phytolacca americana								
2	4194432	QBG Phytolacca japonica		81.64	82.22	83.52				
3	16711680	QBG Talinum crassifolium		77.27	78.17	78.6	79.25	79.65	80.25	81.48
4	13491072	QBG Talinum fruticosum		83						
5	12615680	QBG Talinum paniculatum		77.95	78.87	79.33	79.95	81.28	81.8	83.5
6	16744448	QBG Talinum triangulare		81.3	81.95	83.5				
7	8421376	Other		78.96	78.9	80.25	80.78	81.03	83.4	
8	4227327	Panax ginseng		77.21	77.82	78.09	79.11	81.22	81.75	83.6
9	1677088	Talinum triangulare1		81.2	83.4					
10	16711935	Talinum paniculatum1		83.47	85.3					
11	197379	Talinum triangulare3		81.25	81.98	83.57				
12	13158400	Talinum triangulare2		78.97	80.25	81.1	83.55			
13	8404992	Phytolacca americana1		81.25	81.85	83.63				
14	8510085	Phytolacca americana1		79.62	79.13	80.05	80.7	81.13	81.5	82
15	11893982	Panax ginseng root dry		79.93	80.45	81.27	81.83	83.58		
16	14395776	Panax notoginseng		82.73						
17	14450322	Panax notoginseng root dry		79.03	79.65	80.25	82.77			
18	14515654	dH2O								

No.	Color	Name	Genotype	Peak 43	Peak 44	Peak 45
1	255	QBG Phytolacca americana				
2	4194432	QBG Phytolacca japonica				
3	16711680	QBG Talinum crassifolium		81.83	82.47	84.08
4	13491072	QBG Talinum fruticosum				
5	12615680	QBG Talinum paniculatum				
6	16744448	QBG Talinum triangulare				
7	8421376	Other				
8	4227327	Panax ginseng				
9	1677088	Talinum triangulare1				
10	16711935	Talinum paniculatum1				
11	197379	Talinum triangulare3				
12	13158400	Talinum triangulare2				
13	8404992	Phytolacca americana1				
14	8510085	Phytolacca americana1		83.57		
15	11893982	Panax ginseng root dry				
16	14395776	Panax notoginseng				

17 14450322 Panax notoginseng root dry
18 14515654 dH2O

rbcL(3)

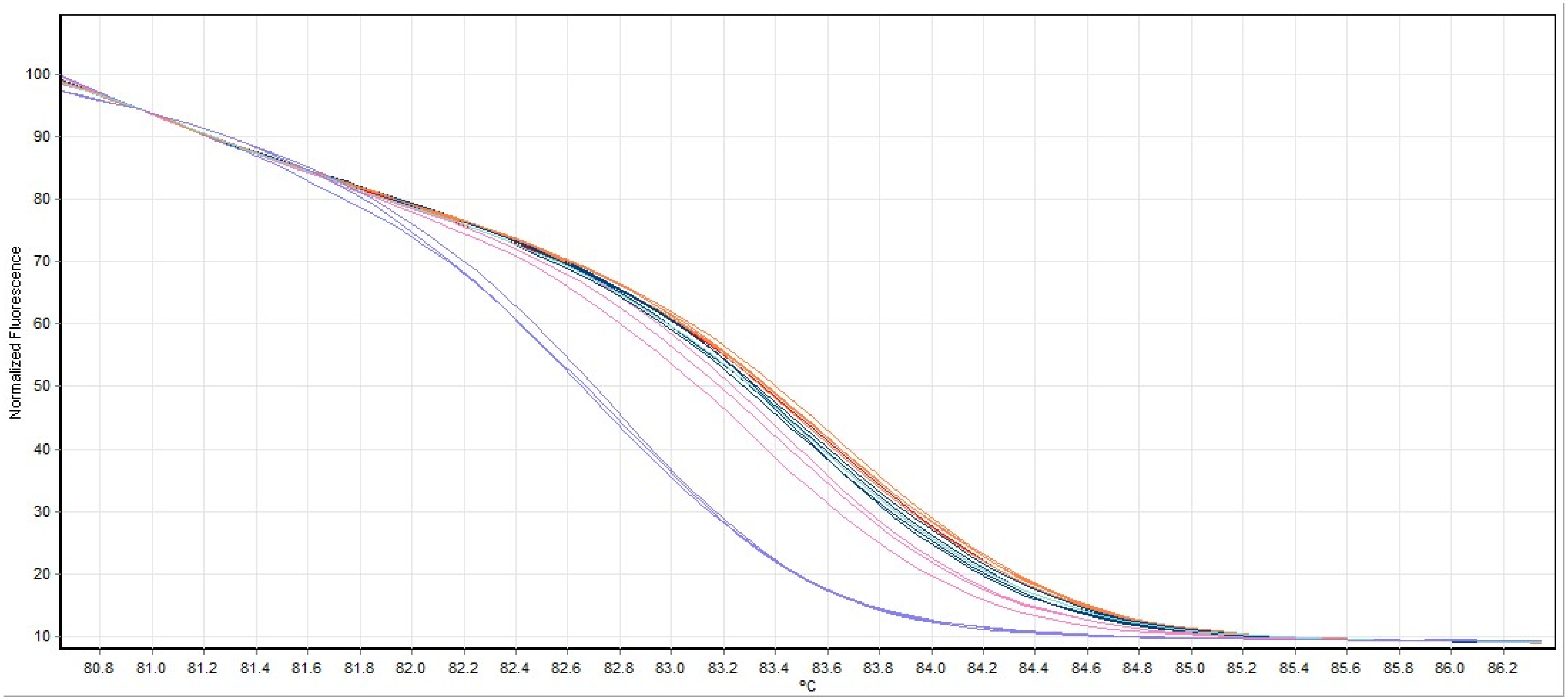

Excel Analysed Data Export
Copyright (c) 2013 QIAGEN GmbH. All Rights Reserved.
File panax HRM_trnL(A).rex

Operator
Run Id
Notes
Machine Serial No 814137

Channel	Gain
Green	5
Yellow	5
Orange	5
Red	5
HRM	-2.66667
Crimson	7

Channel	Threshold

HRM A.HRM (Page 1)

Melt analysis of HRM A.HRM (Page 1)

No.	Color	Name	Genotype	Peak 1	Peak 2	Peak 3	Peak 4	Peak 5	Peak 6	Peak 7
1	4227327	QBG Phytolacca americana		60.85	61.48	62.22	62.77	63.45	63.92	64.48
2	4194432	QBG Phytolacca japonica		60.47	60.95	61.72	62.25	62.7	63.4	63.88
3	16711680	QBGTalinum crassifolium		61.13	61.87	62.35	62.88	63.4	63.83	64.4
4	13491072	QBG Talinum fruticosum		60.9	61.7	62.25	63.03	63.6	64.05	64.55
5	12615680	QBG Talinum paniculatum		60.43	60.88	61.65	62.1	62.55	62.85	63.33
6	8404992	QBG Talinum triangulare		60.57	61.07	61.28	61.7	62.32	62.6	63.05
7	8421376	Other		60.68	61.05	61.73	62.3	62.7	63.47	64.02
8	8421631	Other		60.65	61.07	61.8	62.28	62.85	63.53	63.95
9	1677088	Other		60.25	60.77	61.05	61.52	61.97	62.27	62.82
10	16711935	Other		60.12	60.93	61.5	62.12	62.82	63.4	64.05
11	197379	Other		60.55	60.88	61.3	61.7	62.43	62.93	63.48
12	13158400	Other		60.22	60.65	61.22	61.75	62.28	62.77	63.42
13	8504538	Talinum triangulare2		60.65	61.08	61.55	61.95	62.25	62.77	63.17
14	8510085	Other		60.85	61.25	61.45	62.05	62.77	63.28	63.75
15	11893982	Panax ginseng root dry		60.92	61.25	62.2	62.85	63.35	64.05	64.58
16	14395776	Other		60.9	61.28	61.75	62.42	62.95	63.45	64.05
17	14450322	Panax notoginseng		60.68	61.08	61.72	62.25	62.9	63.48	63.9
18	14515654	Other		60.1	60.7	61.4	61.77	62.25	62.77	63.48
19	11893982	H2O		60.9	61.62	62.05	63.27	64.15	64.67	65.07

No.	Color	Name	Genotype	Peak 8	Peak 9	Peak 10	Peak 11	Peak 12	Peak 13	Peak 14

1	4227327	QBG Phytolacca americana		64.62	65.17	66	66.5	66.85	67.25	67.75
2	4194432	QBG Phytolacca japonica		64.43	64.93	65.42	65.92	66.47	66.85	67.22
3	16711680	QBGTalinum crassifolium		64.97	65.28	65.8	66.12	66.6	66.85	67.43
4	13491072	QBG Talinum fruticosum		65.1	65.55	66.1	66.6	67.12	67.52	68
5	12615680	QBG Talinum paniculatum		63.92	64.55	65.12	65.8	66.15	66.43	66.98
6	8404992	QBG Talinum triangulare		63.42	63.92	64.53	65	65.27	65.77	66.4
7	8421376	Other		64.4	64.88	65.38	65.83	66.48	66.88	67.4
8	8421631	Other		64.43	65.32	66.17	66.88	67.48	68.45	69.1
9	1677088	Other		63.22	63.65	64.1	65.17	65.6	66.15	66.67
10	16711935	Other		64.52	64.98	65.92	67.03	67.85	68.35	68.75
11	197379	Other		64.03	64.35	64.98	65.47	65.8	66.5	67.15
12	13158400	Other		63.93	64.4	64.8	65.23	65.72	66.13	66.52
13	8504538	Talinum triangulare2		63.68	64.45	65.25	65.67	66.15	66.8	67.27
14	8510085	Other		64.18	64.63	65.08	65.68	66.1	66.6	67.05
15	11893982	Panax ginseng root dry		65.18	65.9	66.55	66.95	67.65	68.25	68.65
16	14395776	Other		64.67	65.05	65.38	65.8	66.42	66.88	67.6
17	14450322	Panax notoginseng		64.27	65.3	65.8	66.45	67.45	67.62	68.17
18	14515654	Other		64.25	65.22	65.73	66.12	66.88	67.55	68.12
19	11893982	H2O		65.65	66.1	67.45	68.7	69.75	70.4	71.32

No.	Color	Name	Genotype	Peak 15	Peak 16	Peak 17	Peak 18	Peak 19	Peak 20	Peak 21
1	4227327	QBG Phytolacca americana		68.42	68.68	69.2	69.9	70.37	70.85	71.28
2	4194432	QBG Phytolacca japonica		67.77	68.68	69.08	69.77	70.27	70.95	71.45
3	16711680	QBGTalinum crassifolium		67.73	68.25	69.08	69.72	70.2	70.5	70.97
4	13491072	QBG Talinum fruticosum		68.38	68.85	69.2	69.75	70.43	70.85	71.3
5	12615680	QBG Talinum paniculatum		67.83	68.37	68.75	69.27	69.8	70.25	70.85
6	8404992	QBG Talinum triangulare		66.92	67.65	68.32	68.75	69.38	69.9	70.35
7	8421376	Other		67.9	68.63	69.15	69.65	70.23	70.7	71.05
8	8421631	Other		69.63	70.22	71.1	71.72	72.15	72.77	73.05
9	1677088	Other		67.25	67.6	67.95	68.35	68.73	69.1	69.65
10	16711935	Other		69.32	69.98	70.38	71.07	71.42	71.85	72.25
11	197379	Other		67.8	68.32	68.7	69.25	69.7	70.23	71.1
12	13158400	Other		66.95	67.4	67.78	68.47	68.93	69.2	69.77
13	8504538	Talinum triangulare2		67.75	68.4	69.15	69.75	70.25	70.95	71.85
14	8510085	Other		67.97	68.33	68.75	69.3	69.7	70.35	70.8
15	11893982	Panax ginseng root dry		69.33	70.02	70.4	70.93	71.43	71.95	72.22
16	14395776	Other		67.88	68.67	69.27	70.02	70.47	70.95	71.2

17	14450322	Panax notoginseng		68.55	69.03	69.22	69.65	70.42	70.95	71.55
18	14515654	Other		68.55	69.13	69.57	70.13	70.42	70.85	71.25
19	11893982	H2O		72.1	72.58	73.52	74.52	75.32	75.78	77.05

No.	Color	Name	Genotype	Peak 22	Peak 23	Peak 24	Peak 25	Peak 26	Peak 27	Peak 28
1	4227327	QBG Phytolacca americana		71.77	72.28	73	73.35	73.8	74.25	74.93
2	4194432	QBG Phytolacca japonica		71.8	72.35	72.73	73.25	73.65	74.25	74.98
3	16711680	QBGTalinum crassifolium		71.8	72.32	72.78	73.45	73.75	74.23	74.8
4	13491072	QBG Talinum fruticosum		71.8	72.17	72.92	73.67	74.17	74.52	75
5	12615680	QBG Talinum paniculatum		71.35	71.85	72.2	72.58	72.93	73.52	74.05
6	8404992	QBG Talinum triangulare		71	71.47	71.9	72.37	73.03	73.6	74.25
7	8421376	Other		71.5	71.9	72.37	72.7	73.45	74.25	75.05
8	8421631	Other		73.77	74.22	74.75	75.42	75.65	76.07	76.4
9	1677088	Other		70.13	70.4	70.85	71.2	71.63	72.2	72.9
10	16711935	Other		72.48	72.93	73.4	74.13	74.55	75.75	76.2
11	197379	Other		71.75	72.75	73.2	74.13	74.92	75.73	76.22
12	13158400	Other		70.22	70.68	71	71.5	71.93	72.65	73
13	8504538	Talinum triangulare2		72.25	72.85	73.27	73.8	74.2	74.75	75.37
14	8510085	Other		71.35	71.85	72.23	72.87	73.55	74.18	74.75
15	11893982	Panax ginseng root dry		72.7	73.47	73.93	74.33	74.72	75.48	75.85
16	14395776	Other		71.77	71.92	72.33	72.98	73.82	74.05	74.77
17	14450322	Panax notoginseng		71.8	72.37	73.05	73.55	74.2	74.8	75.55
18	14515654	Other		71.72	72.22	72.9	73.32	73.72	74.25	74.78
19	11893982	H2O		77.85	78.38	78.87	79.62	80.25	80.8	81.22

No.	Color	Name	Genotype	Peak 29	Peak 30	Peak 31	Peak 32	Peak 33	Peak 34	Peak 35
1	4227327	QBG Phytolacca americana		75.45	75.75	76.27	76.8	77.4	77.7	78.2
2	4194432	QBG Phytolacca japonica		75.53	76.13	76.82	77.25	77.73	78.33	79.3
3	16711680	QBGTalinum crassifolium		75.03	75.5	76.02	76.67	77.35	77.7	78.25
4	13491072	QBG Talinum fruticosum		75.4	75.75	76.32	76.95	77.37	77.85	78.35
5	12615680	QBG Talinum paniculatum		74.5	75.7	76.25	77	77.52	78.07	80.4
6	8404992	QBG Talinum triangulare		74.68	75.1	75.45	75.92	76.25	76.75	77.17
7	8421376	Other		75.55	76.17	76.75	77.63	80.85	83.55	84.27
8	8421631	Other		76.7	77.2	77.7	78.25	78.73	81.15	84.37
9	1677088	Other		73.65	74.25	74.5	74.85	75.35	75.92	76.35
10	16711935	Other		76.65	77.23	78	80.27	82.05	84.15	84.85
11	197379	Other		76.8	77.73	80.3	81.95	83.87	84.75	85.35
12	13158400	Other		73.6	74.13	75.1	75.53	76.1	76.9	77.28

13	8504538	Talinum triangulare2		76.2	76.75	77.1	77.87	80.45	82.32	87.87
14	8510085	Other		75.28	75.75	76.25	77.05	77.37	77.92	78.42
15	11893982	Panax ginseng root dry		76.68	77.2	77.6	78	78.4	78.88	79.37
16	14395776	Other		75.02	75.63	76.17	76.78	77.2	77.65	78.08
17	14450322	Panax notoginseng		76.12	76.67	77.17	77.78	78.2	78.65	79.48
18	14515654	Other		75.33	75.73	76.4	76.85	77.63	78.3	79.12
19	11893982	H2O		82.05	83.3	84.08	84.55	84.97	85.48	86.3

No.	Color	Name	Genotype	Peak 36	Peak 37	Peak 38	Peak 39	Peak 40	Peak 41	Peak 42
1	4227327	QBG Phytolacca americana		78.68	81.45					
2	4194432	QBG Phytolacca japonica		81.25	84.2	87.22				
3	16711680	QBGTalinum crassifolium		80.63	82.35	85.23	85.72	86.58	87.05	87.7
4	13491072	QBG Talinum fruticosum		78.7	79.15	81.62	83.35	83.78	84.45	
5	12615680	QBG Talinum paniculatum		82.1						
6	8404992	QBG Talinum triangulare		77.6	80.35	82.25	84.08	84.75	85.2	85.47
7	8421376	Other		84.77	85.22	86.05	86.65	87.33	87.8	88.53
8	8421631	Other		85.17	87.03					
9	1677088	Other		77.05	77.93	80.35	82.03	84.38	85	85.5
10	16711935	Other		85.5	85.9	86.42	86.78	87.08	87.2	87.58
11	197379	Other		85.93	86.43	86.82	87.27	88.45	89.25	
12	13158400	Other		77.85	80.35	82.4	84.3	84.78	85.17	85.65
13	8504538	Talinum triangulare2								
14	8510085	Other		80.45	82.15	82.42	84.48	84.93	85.43	85.85
15	11893982	Panax ginseng root dry		79.82	80.32	80.8	81.25	81.67	83.98	
16	14395776	Other		79.18	79.43	79.85	80.38	81.3	83.67	85.85
17	14450322	Panax notoginseng		80.28	80.7	83.8				
18	14515654	Other		81.15	82.63	85.08	85.8	86.2	86.55	87
19	11893982	H2O		86.8	87.58	88.23	88.67	89.6		

No.	Color	Name	Genotype	Peak 43	Peak 44	Peak 45	Peak 46	Peak 47	Peak 48	Peak 49
1	4227327	QBG Phytolacca americana								
2	4194432	QBG Phytolacca japonica								
3	16711680	QBGTalinum crassifolium		88.5	88.8	89.53				
4	13491072	QBG Talinum fruticosum								
5	12615680	QBG Talinum paniculatum								
6	8404992	QBG Talinum triangulare		85.92	86.37	86.77	87.2	87.7	88.28	88.53
7	8421376	Other		89.2	89.67					
8	8421631	Other								

9	1677088	Other		85.75	86.3	86.97	87.6	88.15	88.8	89.1
10	16711935	Other		88.2	88.65	89.32				
11	197379	Other								
12	13158400	Other		86.05	86.45	86.75	87.25	87.73	88.5	89.05
13	8504538	Talinum triangulare2								
14	8510085	Other		86.18	86.7	87.7	88.25	88.83	89.58	
15	11893982	Panax ginseng root dry								
16	14395776	Other		86.42	86.85	87.18	87.9	88.45	89.23	89.77
17	14450322	Panax notoginseng								
18	14515654	Other		87.6	87.85	88.35	88.95	89.5		
19	11893982	H2O								

No.	Color	Name	Genotype	Peak 50	Peak 51
1	4227327	QBG Phytolacca americana			
2	4194432	QBG Phytolacca japonica			
3	16711680	QBGTalinum crassifolium			
4	13491072	QBG Talinum fruticosum			
5	12615680	QBG Talinum paniculatum			
6	8404992	QBG Talinum triangulare		89.2	89.85
7	8421376	Other			
8	8421631	Other			
9	1677088	Other		89.55	
10	16711935	Other			
11	197379	Other			
12	13158400	Other		89.63	
13	8504538	Talinum triangulare2			
14	8510085	Other			
15	11893982	Panax ginseng root dry			
16	14395776	Other			
17	14450322	Panax notoginseng			
18	14515654	Other			
19	11893982	H2O			

trnL(1)

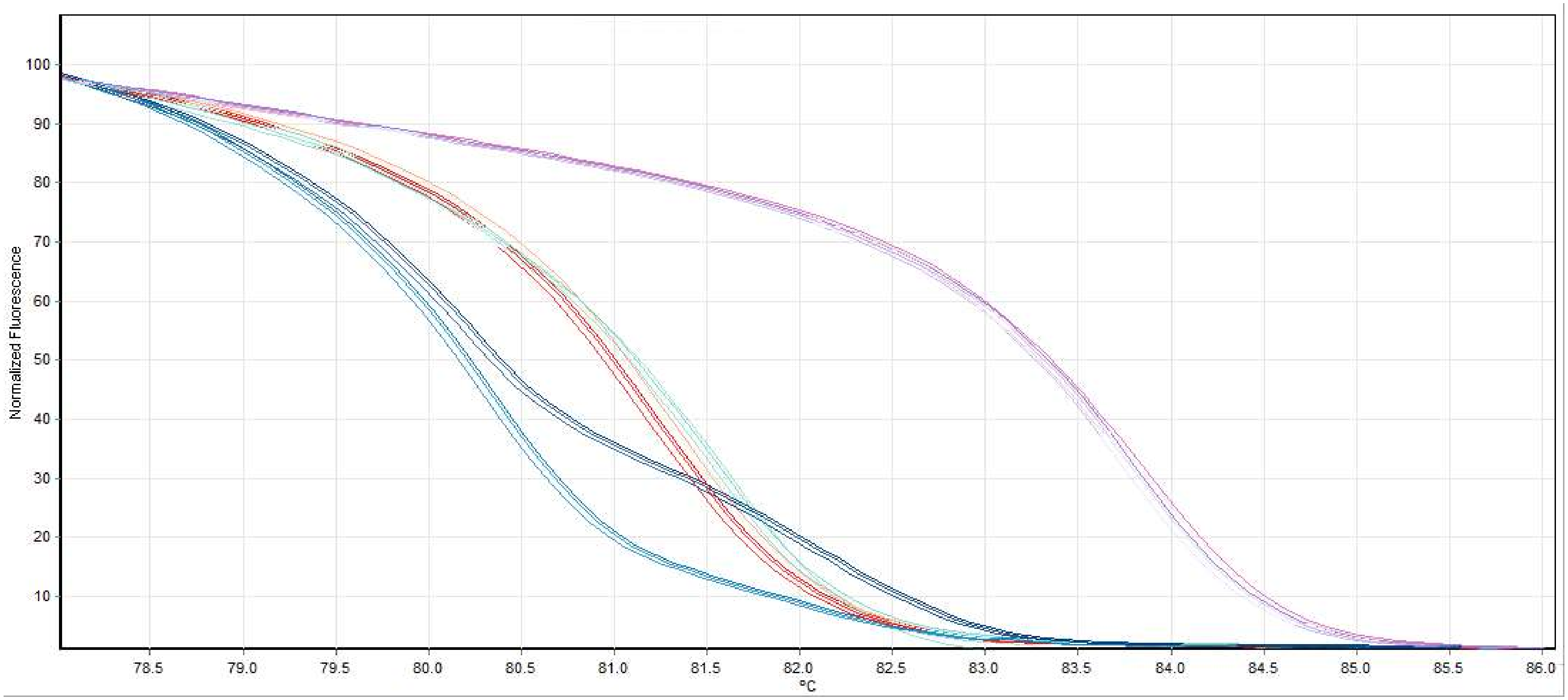

Excel Analysed Data Export

Copyright (c) 2013 QIAGEN GmbH. All Rights Reserved.

File panax HRM_trnL(B).rex

Operator

Run Id

Notes

Machine Serial No 814137

Channel	Gain
Green	5

Yellow	5
Orange	5
Red	5
HRM	-2.66667
Crimson	7

Channel	Threshold
Cycling A.Green (Page 1)	
HRM A.HRM (Page	0.07489

Melt analysis of HRM A.HRM (Page 1)

No.	Color	Name	Genotype	Peak 1	Peak 2	Peak 3	Peak 4	Peak 5	Peak 6	Peak 7
1	255	QBG Phytolacca americana		60.74	61.31	62.1	62.82	63.27	63.81	64.5
2	51400	QBG Phytolacca japonica		60.39	60.75	61.64	62.09	62.62	63.28	63.69
3	16711680	QBGTalinum crassifolium		61.28	61.6	62.19	62.91	63.29	63.74	64.28
4	8388736	QBG Talinum fruticosum		60.84	61.54	62.36	63.14	63.72	64.11	64.62
5	16744703	QBG Talinum paniculatum		60.39	60.92	61.47	62.01	62.49	62.77	63.18
6	16744448	QBG Talinum triangulare		60.67	61.18	61.22	61.65	62.27	62.57	63.14
7	8421376	Other		60.71	61.01	61.87	62.47	62.86	63.42	64.07
8	8421631	Other		60.62	61.14	61.91	62.31	62.91	63.47	63.92
9	1677088	Other		60.34	60.71	61.12	61.66	62	62.34	62.91
10	16711935	Other		60.31	60.87	61.41	62.03	62.93	63.31	63.97
11	197379	Other		60.47	60.92	61.28	61.87	62.31	62.84	63.27
12	13158400	Other		60.17	60.77	61.45	61.82	62.14	62.81	63.35
13	8504538	Talinum triangulare2		60.72	61.14	61.39	61.88	62.28	62.69	63.07
14	8510085	Other		60.92	61.18	61.57	62.14	62.69	63.41	63.82
15	13491072	Panax ginseng root dry		60.97	61.32	62.11	62.92	63.47	64.12	64.36
16	14395776	Other		60.84	61.17	61.82	62.3	62.83	63.28	64.13
17	14450322	Panax notoginseng		60.7	61.1	61.69	62.14	62.79	63.29	63.83
18	14515654	Other		60.25	60.69	61.28	61.82	62.15	62.81	63.52
19	11893982	dH2O								
20	174	dH2O								

No.	Color	Name	Genotype	Peak 8	Peak 9	Peak 10	Peak 11	Peak 12	Peak 13	Peak 14
1	255	QBG Phytolacca americana		64.81	65.29	66.08	66.61	66.92	67.34	67.81

2	51400	QBG Phytolacca japonica		64.29	65	65.48	65.87	66.31	66.92	67.13
3	16711680	QBGTalinum crassifolium		64.89	65.22	65.91	66.23	66.74	66.89	67.25
4	8388736	QBG Talinum fruticosum		65.09	65.62	66.09	66.48	67.19	67.38	68.01
5	16744703	QBG Talinum paniculatum		63.79	64.61	65.08	65.77	66.28	66.49	67.1
6	16744448	QBG Talinum triangulare		63.58	63.97	64.42	65.05	65.19	65.98	66.31
7	8421376	Other		64.52	64.91	65.35	65.92	66.52	66.9	67.37
8	8421631	Other		64.27	65.45	66.28	66.93	67.52	68.6	69.22
9	1677088	Other		63.18	63.75	64.22	65.38	65.76	66.07	66.74
10	16711935	Other		64.47	65.1	65.87	67.01	67.74	68.27	68.83
11	197379	Other		64.11	64.47	65.02	65.42	65.76	66.39	67.09
12	13158400	Other		63.85	64.35	64.92	65.14	65.83	66.07	66.47
13	8504538	Talinum triangulare2		63.54	64.52	65.18	65.56	66.09	66.88	67.18
14	8510085	Other		64.1	64.78	65.24	65.55	66.07	66.74	67.1
15	13491072	Panax ginseng root dry		65.22	66	66.67	66.9	67.49	68.36	68.74
16	14395776	Other		64.57	65.17	65.42	65.78	66.37	66.92	67.53
17	14450322	Panax notoginseng		64.31	65.23	65.77	66.61	67.31	67.67	68.09
18	14515654	Other		64.13	65.09	65.64	66.24	66.75	67.48	68.2
19	11893982	dH2O								
20	174	dH2O								

No.	Color	Name	Genotype	Peak 15	Peak 16	Peak 17	Peak 18	Peak 19	Peak 20	Peak 21
1	255	QBG Phytolacca americana		68.27	68.59	69.27	69.87	70.21	70.76	71.33
2	51400	QBG Phytolacca japonica		67.98	68.42	69.1	69.64	70.18	70.9	71.44
3	16711680	QBGTalinum crassifolium		67.84	68.17	69.13	69.89	70.31	70.64	70.96
4	8388736	QBG Talinum fruticosum		68.42	68.79	69.32	69.81	70.26	70.77	71.19
5	16744703	QBG Talinum paniculatum		67.92	68.29	68.81	69.16	69.74	70.09	70.72
6	16744448	QBG Talinum triangulare		66.79	67.52	68.29	68.81	69.28	69.88	70.4
7	8421376	Other		67.93	68.75	69.08	69.74	70.1	70.88	71.17
8	8421631	Other		69.71	70.28	71.07	71.82	72.09	72.61	73.14
9	1677088	Other		67.32	67.81	67.99	68.37	68.84	69.17	69.7
10	16711935	Other		69.24	69.89	70.41	71.12	71.5	71.97	72.17
11	197379	Other		67.79	68.3	68.89	69.33	69.82	70.14	71.09
12	13158400	Other		66.8	67.22	67.69	68.39	68.88	69.35	69.84
13	8504538	Talinum triangulare2		67.66	68.36	69.2	69.87	70.31	71	71.77
14	8510085	Other		67.86	68.28	68.81	69.22	69.81	70.45	70.82
15	13491072	Panax ginseng root dry		69.34	70.1	70.52	70.77	71.28	71.9	72.17
16	14395776	Other		67.72	68.55	69.34	70	70.25	70.67	71.05

17	14450322	Panax notoginseng		68.41	69	69.38	69.75	70.25	70.73	71.45
18	14515654	Other		68.63	69.05	69.61	70.06	70.35	70.97	71.52
19	11893982	dH2O								
20	174	dH2O								

No.	Color	Name	Genotype	Peak 22	Peak 23	Peak 24	Peak 25	Peak 26	Peak 27	Peak 28
1	255	QBG Phytolacca americana		71.74	72.3	73.04	73.4	73.77	74.31	74.9
2	51400	QBG Phytolacca japonica		71.9	72.3	72.69	73.25	73.71	74.25	74.95
3	16711680	QBGTalinum crassifolium		71.74	72.27	72.87	73.37	73.8	74.25	74.83
4	8388736	QBG Talinum fruticosum		71.69	72.11	72.98	73.52	75.74	76.42	76.89
5	16744703	QBG Talinum paniculatum		71.28	71.69	72.09	72.41	72.99	73.47	74.11
6	16744448	QBG Talinum triangulare		71.02	71.57	71.77	72.44	73.08	73.66	74.19
7	8421376	Other		71.61	71.86	72.4	72.82	73.53	74.21	75.09
8	8421631	Other		73.82	74.27	74.69	75.3	75.71	76.15	76.37
9	1677088	Other		70.24	70.56	70.96	71.12	71.55	72.11	72.92
10	16711935	Other		72.56	72.89	73.29	74.07	74.62	75.62	76.18
11	197379	Other		71.64	72.7	73.2	74.09	74.9	75.7	76.37
12	13158400	Other		70.09	70.78	71.04	71.62	72.01	72.74	73.11
13	8504538	Talinum triangulare2		72.24	72.91	73.36	73.79	74.31	74.67	75.28
14	8510085	Other		71.47	71.91	72.28	72.9	73.61	74.27	74.67
15	13491072	Panax ginseng root dry		72.59	73.41	73.89	74.25	74.67	75.31	75.81
16	14395776	Other		71.82	71.84	72.28	72.88	73.91	74.22	74.69
17	14450322	Panax notoginseng		71.78	72.4	73.11	73.46	74.28	74.67	75.53
18	14515654	Other		71.91	72.31	72.81	73.41	73.69	74.31	74.82
19	11893982	dH2O								
20	174	dH2O								

No.	Color	Name	Genotype	Peak 29	Peak 30	Peak 31	Peak 32	Peak 33	Peak 34	Peak 35
1	255	QBG Phytolacca americana		75.51	75.84	76.09	76.72	77.33	77.68	78.22
2	51400	QBG Phytolacca japonica		75.5	76.2	76.91	77.34	77.78	78.41	79.28
3	16711680	QBGTalinum crassifolium		75.01	75.49	76.09	76.58	77.42	77.67	78.3
4	8388736	QBG Talinum fruticosum		78.66	79.19	81.75				
5	16744703	QBG Talinum paniculatum		74.48	75.63	76.35	77.03	77.48	78.1	80.37
6	16744448	QBG Talinum triangulare		74.73	75.17	75.51	75.9	76.21	76.64	77.11
7	8421376	Other		75.62	76.24	76.8	77.57	80.72		
8	8421631	Other		76.81	77.15	77.68	78.22	78.7	81.2	
9	1677088	Other		73.59	74.36	74.61	74.91	75.41	75.9	76.29
10	16711935	Other		76.61	77.22	78.07	80.3	82		

11	197379	Other		76.74	77.61	80.29	81.88			
12	13158400	Other		73.63	74.22	75.12	75.64	76.19	76.81	77.17
13	8504538	Talinum triangulare2		76.17	76.81	77.07	77.92	80.5	82.28	
14	8510085	Other		75.34	75.67	76.17	77.11	77.44	77.96	78.5
15	13491072	Panax ginseng root dry		76.77	77.24	77.74	78.04	78.37	78.9	79.28
16	14395776	Other		75.14	75.6	76.12	76.8	77.31	77.68	78.05
17	14450322	Panax notoginseng		76.05	76.61	77.09	77.82	78.18	78.71	79.5
18	14515654	Other		75.24	75.65	76.36	76.91	77.61	78.33	79.18
19	11893982	dH2O								
20	174	dH2O								

No.	Color	Name	Genotype	Peak 36	Peak 37	Peak 38	Peak 39	Peak 40	Peak 41
1	255	QBG Phytolacca americana		78.74	81.4				
2	51400	QBG Phytolacca japonica		81.17					
3	16711680	QBGTalinum crassifolium		80.55	82.41				
4	8388736	QBG Talinum fruticosum							
5	16744703	QBG Talinum paniculatum		82.08					
6	16744448	QBG Talinum triangulare		77.56	80.33	82.32			
7	8421376	Other							
8	8421631	Other							
9	1677088	Other		77.12	77.89	80.47	82.1		
10	16711935	Other							
11	197379	Other							
12	13158400	Other		77.9	80.3	82.44			
13	8504538	Talinum triangulare2							
14	8510085	Other		80.41	82.23	82.39			
15	13491072	Panax ginseng root dry		79.79	80.44	80.72	81.29	81.61	83.92
16	14395776	Other		79.2	79.51	79.89	80.4	81.27	83.59
17	14450322	Panax notoginseng		80.08	80.65	83.84			
18	14515654	Other		81.1	82.57				
19	11893982	dH2O							
20	174	dH2O							

trnL(2)

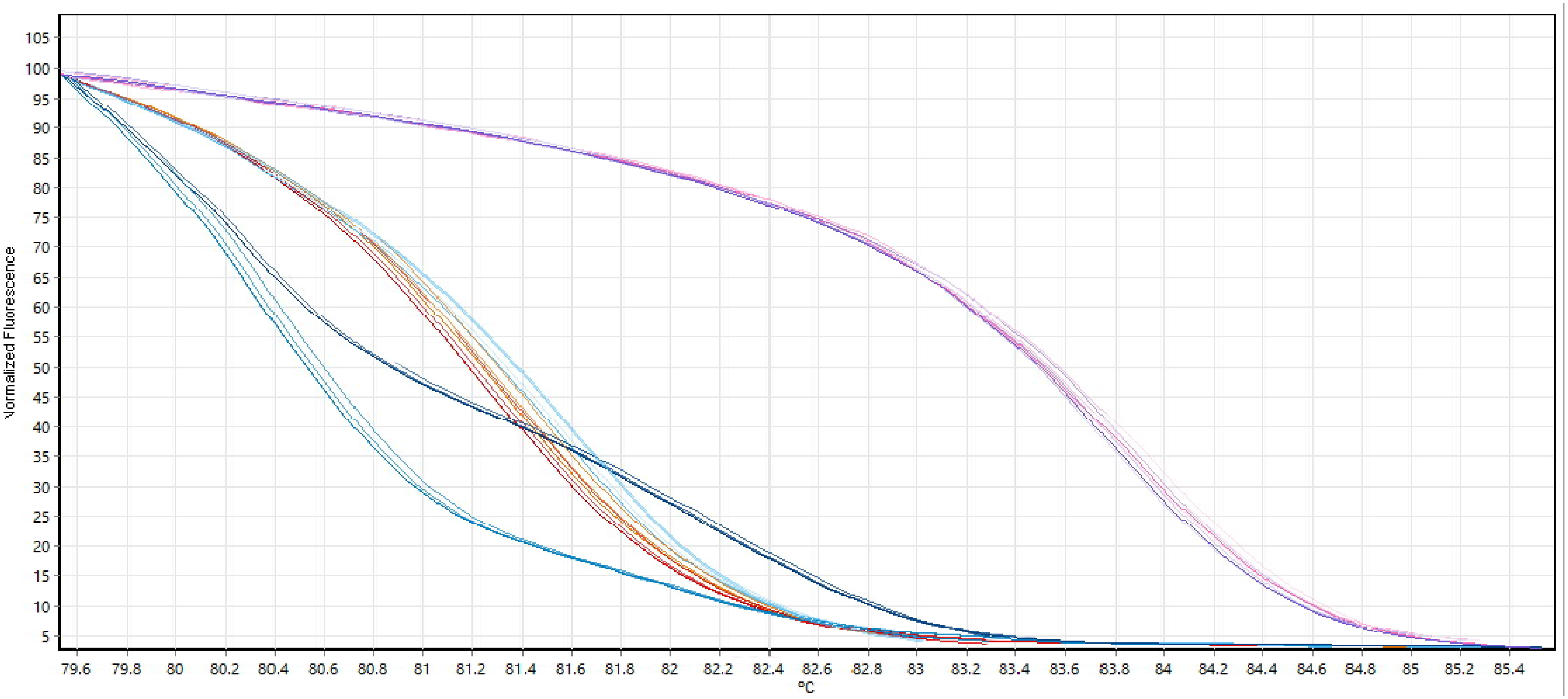

Excel Analysed Data Export
Copyright (c) 2013 QIAGEN GmbH. All Rights Reserved.
File panax HRM_trnL(C).rex

Operator
Run Id
Notes
Machine Serial No 814137

Channel Gain

Green	5
Yellow	5
Orange	5
Red	5
HRM	-2.66667
Crimson	7

Channel	Threshold
Cycling A.Green (Page 1)	
HRM A.HRM (Page	0.12307

Melt analysis of HRM A.HRM (Page 1)

No.	Color	Name	Genotype	Peak 1	Peak 2	Peak 3	Peak 4	Peak 5	Peak 6	Peak 7
1	255	QBG Phytolacca americana		60.8	61.44	62.29	62.76	63.39	63.9	64.41
2	51400	QBG Phytolacca japonica		60.41	60.87	61.77	62.21	62.63	63.44	63.92
3	16711680	QBGTalinum crassifolium		61.07	61.69	62.28	62.74	63.47	63.78	64.38
4	8388736	QBG Talinum fruticosum		60.92	61.72	62.21	63	63.64	64.07	65.17
5	16744703	QBG Talinum paniculatum		60.4	60.85	61.63	62.19	62.62	62.91	63.41
6	16744448	QBG Talinum triangulare		60.66	61.14	61.37	61.74	62.35	62.72	63.09
7	8421376	Other		60.61	61.08	61.68	62.27	62.77	63.5	64.08
8	8421631	Other		60.62	61.05	61.84	62.26	62.8	63.58	63.89
9	1677088	Other		60.17	60.79	61.11	61.59	61.89	62.22	62.87
10	16711935	Other		60.22	60.9	61.43	62.2	62.81	63.31	64.01
11	197379	Other		60.48	60.75	61.32	61.74	62.44	62.91	63.42
12	13158400	Other		60.27	60.66	61.2	61.82	62.32	62.7	63.36
13	8504538	Talinum triangulare2		60.68	61.16	61.59	61.97	62.21	62.72	63.19
14	8510085	Other		60.89	61.21	61.38	62.11	62.8	63.32	63.66
15	13491072	Panax ginseng root dry		60.9	61.29	62.23	62.74	63.28	64.14	64.6
16	14395776	Other		60.9	61.34	61.81	62.51	62.97	63.37	64.02
17	14450322	Panax notoginseng		60.72	61.02	61.79	62.3	62.88	63.41	63.89
18	14515654	Other		60.12	60.67	61.51	61.72	62.29	62.72	63.45
19	11893982	dH2O								
20	174	dH2O								

No.	Color	Name	Genotype	Peak 8	Peak 9	Peak 10	Peak 11	Peak 12	Peak 13	Peak 14

1	255	QBG Phytolacca americana		64.6	65.31	66.04	66.48	66.88	67.19	67.82
2	51400	QBG Phytolacca japonica		64.47	64.97	65.51	65.97	66.56	66.86	67.17
3	16711680	QBGTalinum crassifolium		64.82	65.34	65.83	66.23	66.61	66.89	67.38
4	8388736	QBG Talinum fruticosum		66.19	81.66					
5	16744703	QBG Talinum paniculatum		63.94	64.63	65.17	65.74	66.09	66.4	67.02
6	16744448	QBG Talinum triangulare		63.46	63.9	64.48	65.02	65.31	65.76	66.36
7	8421376	Other		64.41	64.79	65.24	65.87	66.42	66.81	67.41
8	8421631	Other		64.45	65.24	66.09	66.76	67.46	68.57	69.17
9	1677088	Other		63.18	63.67	64.12	65.19	65.63	66.24	66.69
10	16711935	Other		64.47	64.85	65.97	67.11	67.74	68.33	68.72
11	197379	Other		64	64.27	64.85	65.35	65.77	66.56	67.22
12	13158400	Other		63.88	64.41	64.84	65.31	65.8	66.11	66.43
13	8504538	Talinum triangulare2		63.71	64.41	65.31	65.72	66.27	66.97	67.31
14	8510085	Other		64.07	64.67	65.01	65.57	66.08	67.14	67.84
15	13491072	Panax ginseng root dry		65.15	65.85	66.5	66.98	67.67	68.37	68.72
16	14395776	Other		64.64	65.08	65.44	65.81	66.52	66.91	67.61
17	14450322	Panax notoginseng		64.19	65.37	65.87	66.42	67.37	67.58	68.2
18	14515654	Other		64.37	65.26	65.71	66.07	66.91	67.54	68.2
19	11893982	dH2O								
20	174	dH2O								

No.	Color	Name	Genotype	Peak 15	Peak 16	Peak 17	Peak 18	Peak 19	Peak 20	Peak 21
1	255	QBG Phytolacca americana		68.36	68.69	69.24	69.91	70.32	70.88	71.31
2	51400	QBG Phytolacca japonica		67.74	68.7	69.07	69.76	70.23	70.9	71.44
3	16711680	QBGTalinum crassifolium		67.73	68.31	69.11	69.82	70.96	71.87	74.26
4	8388736	QBG Talinum fruticosum								
5	16744703	QBG Talinum paniculatum		67.77	68.4	68.79	69.14	69.77	70.2	70.91
6	16744448	QBG Talinum triangulare		66.95	67.71	68.28	68.79	69.42	69.89	70.38
7	8421376	Other		67.89	68.59	69.11	69.61	70.16	70.74	71.14
8	8421631	Other		69.68	70.37	71.15	71.87	72.18	72.8	73.09
9	1677088	Other		67.31	67.54	67.9	68.32	68.79	69.12	69.67
10	16711935	Other		69.31	69.93	70.32	71.01	71.41	71.75	72.17
11	197379	Other		67.82	68.27	68.77	69.2	69.7	70.26	71.14
12	13158400	Other		66.97	67.41	67.69	68.52	68.96	69.18	69.72
13	8504538	Talinum triangulare2		67.68	68.48	69.23	69.69	70.22	70.93	71.9
14	8510085	Other		80.34	82.09	82.48				
15	13491072	Panax ginseng root dry		69.39	70.14	70.45	70.97	71.4	71.93	72.16

16	14395776	Other		67.79	68.55	69.3	70.08	70.45	70.94	71.22
17	14450322	Panax notoginseng		68.56	69.07	69.28	69.72	70.39	70.93	71.59
18	14515654	Other		68.59	69.1	69.64	70.1	70.38	70.82	71.3
19	11893982	dH2O								
20	174	dH2O								

No.	Color	Name	Genotype	Peak 22	Peak 23	Peak 24	Peak 25	Peak 26	Peak 27	Peak 28
1	255	QBG Phytolacca americana		71.76	72.21	73.01	73.33	73.87	74.17	74.9
2	51400	QBG Phytolacca japonica		71.83	72.34	72.73	73.28	73.62	74.22	74.92
3	16711680	QBGTalinum crassifolium		76	78.2	80.58	82.41			
4	8388736	QBG Talinum fruticosum								
5	16744703	QBG Talinum paniculatum		71.38	71.91	72.14	73.54	74.01	74.57	75.66
6	16744448	QBG Talinum triangulare		71.01	71.39	71.91	72.4	73.08	73.58	74.28
7	8421376	Other		71.49	71.95	72.36	72.67	73.39	74.31	75.11
8	8421631	Other		73.78	74.28	74.69	75.45	75.71	76.11	76.41
9	1677088	Other		70.14	70.44	70.87	71.17	71.66	72.23	72.92
10	16711935	Other		72.97	73.51	74.07	74.51	75.74	76.58	77.22
11	197379	Other		71.69	72.87	74.08	74.9	75.75	76.24	76.79
12	13158400	Other		70.26	70.71	71.02	71.52	71.89	72.69	73.04
13	8504538	Talinum triangulare2		72.27	72.92	73.34	73.86	74.17	74.7	75.47
14	8510085	Other								
15	13491072	Panax ginseng root dry		72.8	73.38	73.9	74.38	74.79	75.41	75.82
16	14395776	Other		71.77	71.9	72.29	72.97	73.88	74.09	74.71
17	14450322	Panax notoginseng		71.78	72.31	73.1	73.48	74.18	74.89	75.57
18	14515654	Other		71.82	72.18	72.91	73.28	73.7	74.2	74.82
19	11893982	dH2O								
20	174	dH2O								

No.	Color	Name	Genotype	Peak 29	Peak 30	Peak 31	Peak 32	Peak 33	Peak 34	Peak 35
1	255	QBG Phytolacca americana		75.52	75.77	76.22	76.84	77.42	77.63	78.14
2	51400	QBG Phytolacca japonica		75.54	76.07	76.78	77.15	77.74	78.41	79.28
3	16711680	QBGTalinum crassifolium								
4	8388736	QBG Talinum fruticosum								
5	16744703	QBG Talinum paniculatum		76.2	76.98	77.63	78.18	80.437	82.17	
6	16744448	QBG Talinum triangulare		74.77	75.11	75.4	76	76.3	76.84	77.24
7	8421376	Other		75.62	76.14	76.81	77.57	80.91		
8	8421631	Other		76.7	77.17	77.64	78.21	78.77	81.27	
9	1677088	Other		73.68	74.21	74.55	74.78	75.42	75.9	76.39

10	16711935	Other		78.04	80.33	82.04				
11	197379	Other		77.77	80.37	81.99				
12	13158400	Other		73.61	74.1	75.17	75.51	76.11	76.95	77.31
13	8504538	Talinum triangulare2		76.19	76.77	77.14	77.81	80.47	82.37	
14	8510085	Other								
15	13491072	Panax ginseng root dry		76.66	77.21	77.68	78.04	78.82	79.8	80.27
16	14395776	Other		75.08	75.73	76.12	76.8	77.15	77.71	78.11
17	14450322	Panax notoginseng		76.14	76.74	77.23	77.77	78.18	78.66	79.6
18	14515654	Other		75.28	75.81	76.41	76.8	77.64	78.3	79.07
19	11893982	dH2O								
20	174	dH2O								

No.	Color	Name	Genotype	Peak 36	Peak 37	Peak 38	Peak 39	Peak 40	Peak 41
1	255	QBG Phytolacca americana		78.71	81.51				
2	51400	QBG Phytolacca japonica		81.31					
3	16711680	QBGTalinum crassifolium							
4	8388736	QBG Talinum fruticosum							
5	16744703	QBG Talinum paniculatum							
6	16744448	QBG Talinum triangulare		77.63	80.45	82.27			
7	8421376	Other							
8	8421631	Other							
9	1677088	Other		77.09	77.94	80.44	82.02		
10	16711935	Other							
11	197379	Other							
12	13158400	Other		77.87	80.36	82.45			
13	8504538	Talinum triangulare2							
14	8510085	Other							
15	13491072	Panax ginseng root dry		80.81	81.19	81.69	83.94		
16	14395776	Other		79.12	79.51	79.81	80.3	81.28	83.69
17	14450322	Panax notoginseng		80.19	80.72	83.78			
18	14515654	Other		81.09	82.64				
19	11893982	dH2O							
20	174	dH2O							

trnL(3)

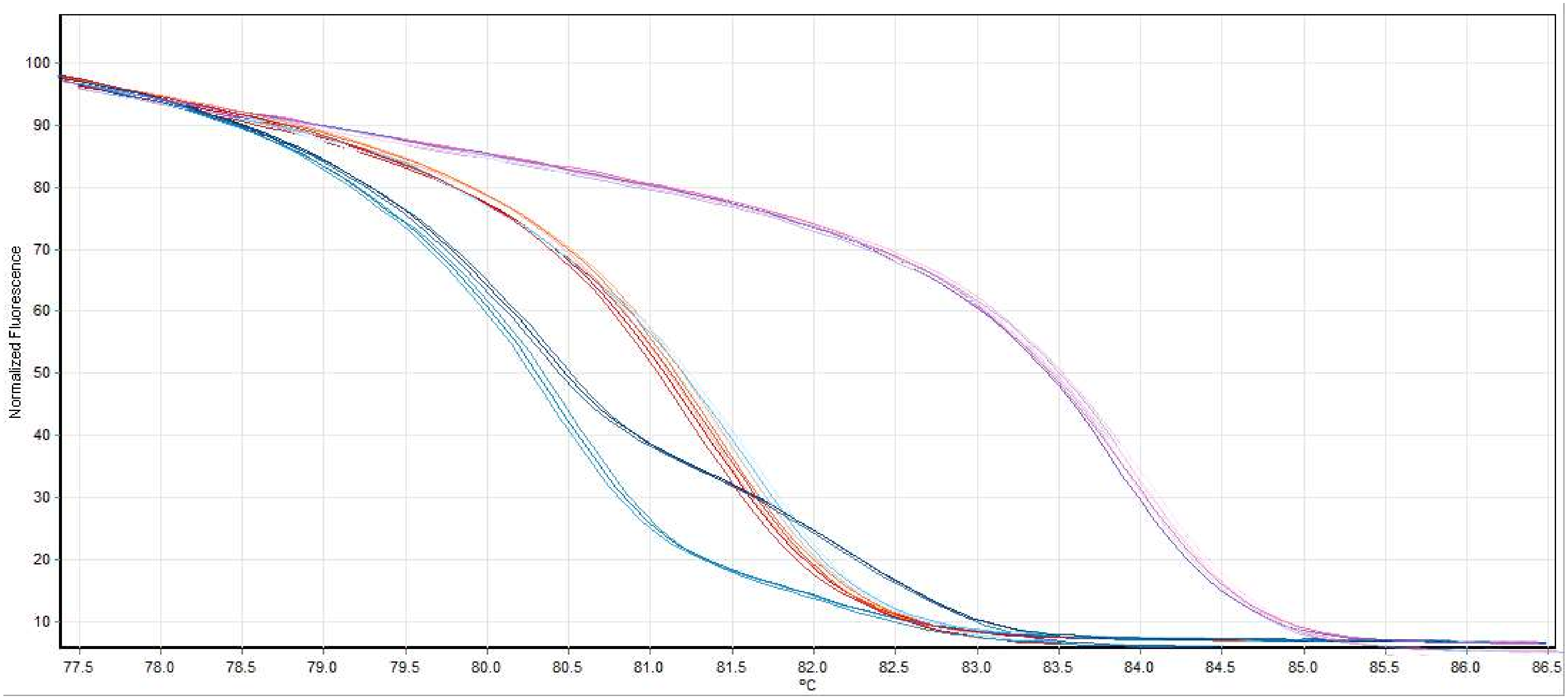

Excel Analysed Data Export
Copyright (c) 2013 QIAGEN GmbH. All Rights Reserved.
File panax HRM_ITS2(1).rex

Operator
Run Id
Notes
Machine Serial No 814137

Channel Gain

Green	5
Yellow	5
Orange	5
Red	5
HRM	-2.33333
Crimson	7

Channel	Threshold

HRM A.HRM (Page	0

Melt analysis of HRM A.HRM (Page 1)

No.	Color	Name	Genotype	Peak 1	Peak 2	Peak 3	Peak 4	Peak 5	Peak 6	Peak 7
1	255	QBG phytolacca americana		60.87	61.2	61.75	62.07	62.35	62.9	63.7
2	51400	QBG phytolacca japonica		60.93	61.45	61.95	62.72	63.53	64.05	64.5
3	16711680	QBG talinum crassifolium		60.65	61.4	61.8	62.2	62.97	63.6	64.05
4	8388736	QBG talinum fruticosum		60.53	61.15	61.75	62.13	62.45	62.9	63.27
5	16744703	QBG talinum paniculatum		60.35	60.88	61.15	61.68	62.5	63	63.75
6	16744448	QBG talinum triangulare		60.17	60.87	61.37	61.6	61.98	62.62	63.25
7	8421376	Other		60.93	61.4	61.9	62.15	62.73	63.45	64.03
8	8421631	phytolacca japonica		60.85	61.55	62.2	62.83	63.7	64.12	64.55
9	1677088	Other		60.72	61.2	61.8	62.92	63.85	64.27	64.93
10	16711935	Other		60.15	60.78	61.13	61.7	62.1	62.52	63.15
11	197379	Other		60.73	61.45	61.67	62.15	62.7	63.2	63.6
12	13158400	Other		60.87	61.45	61.82	62.17	62.72	63.5	64.05
13	8504538	Other		60.57	60.95	61.32	61.75	62.52	62.9	63.4
14	8510085	phytolacca americana dry		60.25	60.5	61.12	61.75	62.28	62.95	63.75
15	13491072	panax ginseng dry		60.25	61.12	61.68	62.03	62.3	62.55	63.13
16	14395776	Other		60.12	60.67	61.55	61.9	62.7	63.5	64.05
17	14450322	panax notoginseng		60.95	61.43	61.9	62.05	62.77	63.35	64.03
18	14515654	phytolacca americana		60.65	61.2	61.75	62.25	62.85	63.65	64.32
19	11893982	dH2O		60.43	60.75	61.18	61.72	62.02	62.45	63.05

No.	Color	Name	Genotype	Peak 8	Peak 9	Peak 10	Peak 11	Peak 12	Peak 13	Peak 14
1	255	QBG phytolacca americana		64.25	64.65	65.02	65.7	66.15	66.62	67.3
2	51400	QBG phytolacca japonica		65.13	65.57	66.25	66.62	67.1	67.65	68.33

3	16711680	QBG talinum crassifolium		64.65	65.25	65.6	66.2	66.6	67.15	67.67
4	8388736	QBG talinum fruticosum		63.73	64.25	64.97	66	66.7	67.3	67.75
5	16744703	QBG talinum paniculatum		64.22	65.1	65.75	66.15	66.4	66.85	67.33
6	16744448	QBG talinum triangulare		63.67	64.03	64.48	64.98	65.47	66.08	66.62
7	8421376	Other		64.45	64.88	65.28	65.58	66.3	66.55	66.77
8	8421631	phytolacca japonica		65.47	66.2	66.75	67.2	67.65	68.35	68.92
9	1677088	Other		65.37	65.85	66.33	66.68	67.3	68.02	68.6
10	16711935	Other		63.73	64.15	64.97	65.4	66.17	66.77	67.32
11	197379	Other		64.07	64.58	64.98	65.35	65.8	66.17	66.63
12	13158400	Other		64.5	65.33	65.83	66.2	66.6	67.12	67.68
13	8504538	Other		63.93	64.15	64.58	65.08	65.82	66.58	67.2
14	8510085	phytolacca americana dry		64.3	65.05	65.8	66.1	66.73	67.38	67.67
15	13491072	panax ginseng dry		63.7	64.23	65.1	65.73	66.1	66.55	66.75
16	14395776	Other		64.55	64.95	65.35	65.73	66.55	67	67.1
17	14450322	panax notoginseng		64.58	65.2	65.65	66.2	66.57	67.15	67.67
18	14515654	phytolacca americana		64.52	64.85	64.97	65.25	65.78	66.12	66.68
19	11893982	dH2O		63.82	64.28	64.9	65.45	65.87	66.3	66.85

No.	Color	Name	Genotype	Peak 15	Peak 16	Peak 17	Peak 18	Peak 19	Peak 20	Peak 21
1	255	QBG phytolacca americana		67.65	67.93	68.38	69	69.35	69.73	70.33
2	51400	QBG phytolacca japonica		68.85	69.35	70.13	70.53	71.07	71.72	72.75
3	16711680	QBG talinum crassifolium		68.33	68.8	69.5	70.45	70.82	71.25	72.05
4	8388736	QBG talinum fruticosum		67.97	68.6	69.1	69.5	70.3	70.87	71.42
5	16744703	QBG talinum paniculatum		68.15	68.63	69.22	69.98	70.65	71.13	71.5
6	16744448	QBG talinum triangulare		67.25	68.12	69.25	69.87	70.6	71.25	71.83
7	8421376	Other		67.15	67.58	68.25	68.95	69.53	70.57	71.27
8	8421631	phytolacca japonica		69.5	70.35	70.75	71.3	72.02	72.75	73.25
9	1677088	Other		69.15	69.5	70.2	70.75	71.42	72.1	72.57
10	16711935	Other		67.72	68.17	68.63	69.15	69.9	70.27	70.87
11	197379	Other		67.08	68.05	68.32	68.97	69.35	70.1	70.95
12	13158400	Other		68.3	68.97	69.5	70.35	70.95	71.3	71.73
13	8504538	Other		67.72	68.42	68.98	69.48	70.52	70.95	71.32
14	8510085	phytolacca americana dry		68.58	69.2	69.88	70.17	70.7	71.45	72.1
15	13491072	panax ginseng dry		67.22	67.55	68.17	68.63	69.25	69.95	70.53
16	14395776	Other		67.55	67.93	68.37	68.97	69.38	69.85	70.77
17	14450322	panax notoginseng		68.17	68.88	69.52	70.3	70.55	71.2	71.75
18	14515654	phytolacca americana		67	67.3	67.7	68.05	68.53	69.05	69.75

19	11893982	dH2O		67.35	67.77	68.2	68.7	69.15	69.9	70.75

No.	Color	Name	Genotype	Peak 22	Peak 23	Peak 24	Peak 25	Peak 26	Peak 27	Peak 28
1	255	QBG phytolacca americana		70.83	71.23	72.05	72.97	73.55	74.35	75.05
2	51400	QBG phytolacca japonica		73.17	73.45	74.13	74.62	75.12	75.7	76.22
3	16711680	QBG talinum crassifolium		72.43	73.07	73.55	74.15	74.55	75.35	75.63
4	8388736	QBG talinum fruticosum		72.07	72.73	73.67	74.6	75.3	76.15	76.75
5	16744703	QBG talinum paniculatum		71.8	72.05	72.45	72.92	73.38	73.9	74.28
6	16744448	QBG talinum triangulare		72.47	72.85	73.3	73.93	74.42	75.03	75.52
7	8421376	Other		71.77	72.5	72.85	73.47	74.2	75.08	75.48
8	8421631	phytolacca japonica		73.57	74.15	74.3	75.02	75.4	75.98	76.45
9	1677088	Other		73.1	73.67	74.33	74.75	75.3	75.83	76.43
10	16711935	Other		71.82	72.25	72.78	73.87	74.7	75.47	76.05
11	197379	Other		71.3	71.78	72.65	73.25	73.72	74.25	74.6
12	13158400	Other		72.1	72.5	73.05	73.45	74.15	74.47	75
13	8504538	Other		72.05	72.4	72.8	73.52	74.3	74.85	75.22
14	8510085	phytolacca americana dry		72.8	73.25	73.82	74.7	75.35	76	76.8
15	13491072	panax ginseng dry		71.28	71.85	72.6	73.1	73.9	74.37	74.75
16	14395776	Other		71.3	71.75	72.45	73.32	73.9	74.25	74.75
17	14450322	panax notoginseng		72.4	73.45	74.15	74.65	75.1	75.63	76.23
18	14515654	phytolacca americana		70.37	71.22	71.5	72.07	72.5	73.08	73.62
19	11893982	dH2O		71.37	71.87	72.77	73.85	74.47	74.8	75.33

No.	Color	Name	Genotype	Peak 29	Peak 30	Peak 31	Peak 32	Peak 33	Peak 34	Peak 35
1	255	QBG phytolacca americana		75.42	75.95	76.43	76.83	77.3	77.95	78.3
2	51400	QBG phytolacca japonica		77.05	77.55	78	78.52	78.78	79.28	79.57
3	16711680	QBG talinum crassifolium		76.42	77.15	77.58	78	78.35	78.87	79.18
4	8388736	QBG talinum fruticosum		77.27	77.83	78.3	78.6	79.4	80.15	80.63
5	16744703	QBG talinum paniculatum		74.73	75.42	76.12	76.7	77.72	78.22	78.53
6	16744448	QBG talinum triangulare		76.15	76.62	77.02	77.52	78.15	78.75	79.48
7	8421376	Other		76.25	77.1	77.58	78.03	78.52	79.05	79.7
8	8421631	phytolacca japonica		77.17	77.93	78.45	79.17	79.95	80.77	81.43
9	1677088	Other		76.75	77.53	77.8	78.33	78.65	79.4	79.93
10	16711935	Other		76.7	76.82	77.47	77.75	78.35	78.95	79.43
11	197379	Other		75.15	75.7	76.1	76.53	77.07	77.68	78.17
12	13158400	Other		75.43	75.72	76.22	76.97	77.63	77.97	78.5
13	8504538	Other		75.65	76.45	77.13	77.75	78	78.4	78.88
14	8510085	phytolacca americana dry		77.4	77.85	78.37	78.7	79.42	80.12	80.78

15	13491072	panax ginseng dry		75.45	76.03	76.77	77.37	78.05	78.95	79.52
16	14395776	Other		75.08	75.7	76.18	76.57	77	77.45	78.18
17	14450322	panax notoginseng		76.63	77.1	77.6	77.98	78.43	78.9	79.2
18	14515654	phytolacca americana		74.3	74.87	75.35	75.82	76.62	77.17	77.83
19	11893982	dH2O		75.97	76.72	77.42	77.83	78.3	78.85	79.42

No.	Color	Name	Genotype	Peak 36	Peak 37	Peak 38	Peak 39	Peak 40	Peak 41	Peak 42
1	255	QBG phytolacca americana		78.87	79.17	79.65	80.4	81	81.32	81.73
2	51400	QBG phytolacca japonica		80.05	80.42	81.05	81.93	82.78	83.42	83.85
3	16711680	QBG talinum crassifolium		79.6	80.35	80.75	81.2	81.87	82.32	82.75
4	8388736	QBG talinum fruticosum		81.2	81.5	82.4	83.17	83.63	84	84.35
5	16744703	QBG talinum paniculatum		78.97	79.45	80.12	80.52	81.05	81.43	81.8
6	16744448	QBG talinum triangulare		80.4	81.1	81.7	82.13	82.83	83.12	83.7
7	8421376	Other		80.4	81.1	82.05	82.75	83.07	83.53	84.05
8	8421631	phytolacca japonica		82.33	82.78	84.52	85.37	85.98	86.72	87.07
9	1677088	Other		80.13	80.65	80.85	81.35	82	82.42	83.27
10	16711935	Other		80.1	80.7	81.63	82.05	82.5	83.43	84.05
11	197379	Other		78.75	79.2	79.45	79.95	80.37	81.07	81.78
12	13158400	Other		79.08	79.6	80.07	80.55	80.67	81.13	81.58
13	8504538	Other		79.58	80	80.8	81.35	81.88	82.33	82.73
14	8510085	phytolacca americana dry		81.33	81.82	82.52	82.95	83.47	83.95	84.38
15	13491072	panax ginseng dry		79.95	80.27	80.72	81.05	81.4	82.17	82.65
16	14395776	Other		78.73	79.3	80.02	80.55	81.05	81.73	82.15
17	14450322	panax notoginseng		79.67	80.5	80.93	81.45	81.95	82.35	83
18	14515654	phytolacca americana		78.3	78.92	79.55	79.95	80.75	81.3	81.9
19	11893982	dH2O		79.85	80.13	80.57	81.35	82.45	82.9	83.25

No.	Color	Name	Genotype	Peak 43	Peak 44	Peak 45	Peak 46	Peak 47	Peak 48	Peak 49
1	255	QBG phytolacca americana		82.4	82.8	83.35	83.7	84.2	84.45	85.07
2	51400	QBG phytolacca japonica		84.2	84.87	85.37	85.8	86.23	86.75	87.92
3	16711680	QBG talinum crassifolium		83.45	83.75	84.63	85.15	85.72	86.15	86.68
4	8388736	QBG talinum fruticosum		87.67						
5	16744703	QBG talinum paniculatum		82.53	82.97	83.52	84.05	84.55	84.83	85.3
6	16744448	QBG talinum triangulare		84.17	84.9	85.35	85.85	86.33	86.78	87.95
7	8421376	Other		84.78	85.25	85.65	86.12	86.68	87.1	87.95
8	8421631	phytolacca japonica		88.33	88.77	89.65				
9	1677088	Other		83.65	84.35	84.85	85.95	86.4	87.25	87.72
10	16711935	Other		84.52	84.92	85.15	85.98	86.47	86.9	87.6

11	197379	Other		82.05	82.83	83.67	84.07	84.82	85.35	85.82
12	13158400	Other		82.02	82.35	82.78	83.1	83.9	84.75	85.23
13	8504538	Other		83.47	84.23	84.63	85.08	86.17	86.65	87.17
14	8510085	phytolacca americana dry		84.8	85.13	85.55	86.37	86.8	87.3	87.6
15	13491072	panax ginseng dry		83.55	84.15	85.25	85.63	86	86.38	87.68
16	14395776	Other		82.85	83.35	83.92	84	84.65	84.9	85.35
17	14450322	panax notoginseng		83.48	83.87	84.33	84.65	85.23	85.67	86.08
18	14515654	phytolacca americana		82.35	82.75	83.33	84.37	85.15	85.55	85.92
19	11893982	dH2O		83.63	84.05	84.45	85.1	85.55	85.98	86.4

No.	Color	Name	Genotype	Peak 50	Peak 51	Peak 52	Peak 53	Peak 54	Peak 55	Peak 56
1	255	QBG phytolacca americana		85.93	86.85	88.05	88.3	88.98	89.63	
2	51400	QBG phytolacca japonica		88.42	89.35					
3	16711680	QBG talinum crassifolium		87.07	87.58	87.85	88.4			
4	8388736	QBG talinum fruticosum								
5	16744703	QBG talinum paniculatum		86.02	86.5	86.85	87.35	87.52	88.17	88.9
6	16744448	QBG talinum triangulare		88.87	89.28					
7	8421376	Other		88.45	89.15	89.6				
8	8421631	phytolacca japonica								
9	1677088	Other		88.23	88.7	89.07	89.63			
10	16711935	Other		88.1	88.87					
11	197379	Other		86.32	86.9	87.12	87.42	87.95	88.38	88.83
12	13158400	Other		85.72	86.2	87.93	88.43	89.32		
13	8504538	Other		87.85	88.35	89.17				
14	8510085	phytolacca americana dry		88.12	88.57	88.83	89.5			
15	13491072	panax ginseng dry		88.2						
16	14395776	Other		85.8	86.3	86.65	87.15	87.95	88.43	89.15
17	14450322	panax notoginseng		86.68	87.85	88.32	89.03	89.48		
18	14515654	phytolacca americana		86.95	87.75	88.73				
19	11893982	dH2O		86.85	87.3	87.6	88.1	88.58	89.05	

No.	Color	Name	Genotype	Peak 57
1	255	QBG phytolacca americana		
2	51400	QBG phytolacca japonica		
3	16711680	QBG talinum crassifolium		
4	8388736	QBG talinum fruticosum		
5	16744703	QBG talinum paniculatum		89.28
6	16744448	QBG talinum triangulare		

7	8421376	Other	
8	8421631	phytolacca japonica	
9	1677088	Other	
10	16711935	Other	
11	197379	Other	89.25
12	13158400	Other	
13	8504538	Other	
14	8510085	phytolacca americana dry	
15	13491072	panax ginseng dry	
16	14395776	Other	89.55
17	14450322	panax notoginseng	
18	14515654	phytolacca americana	
19	11893982	dH2O	

ITS(1)

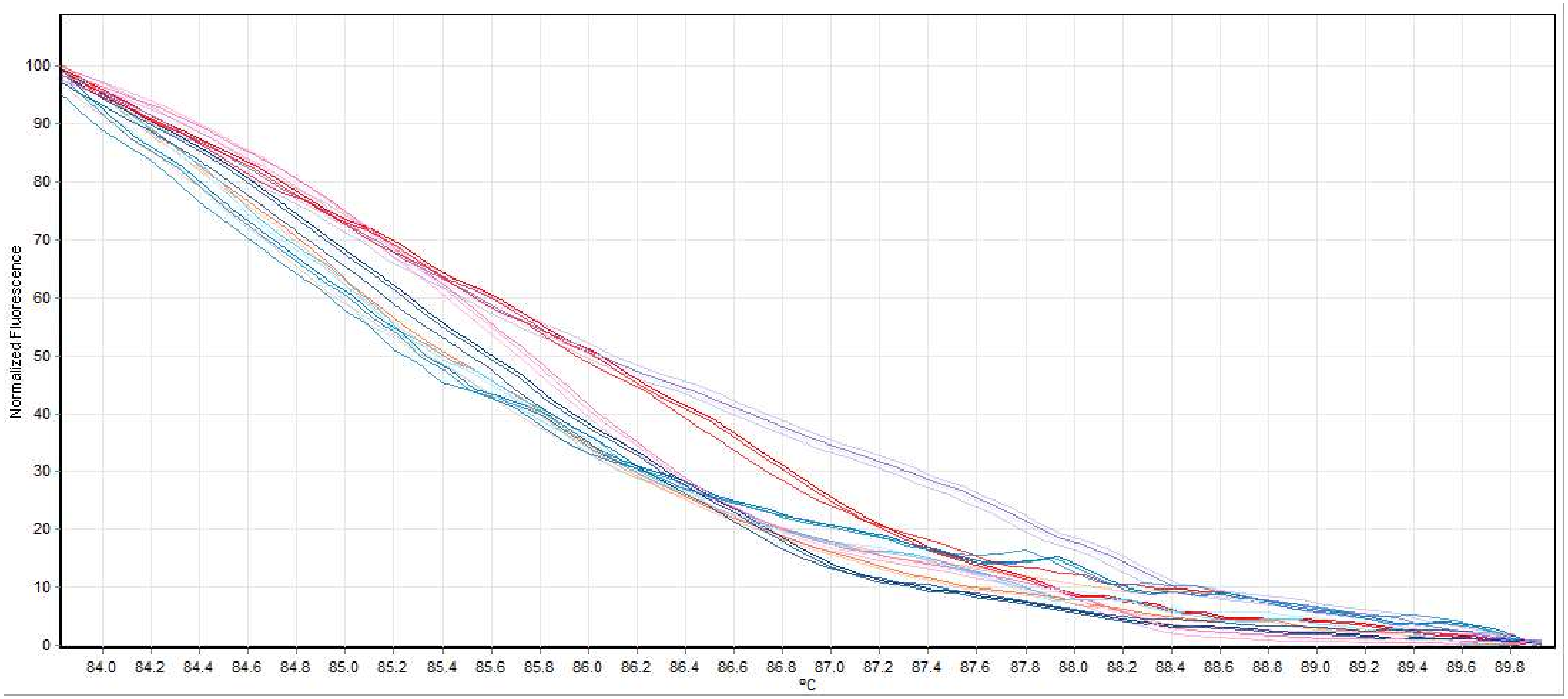

Excel Analysed Data Export
Copyright (c) 2013 QIAGEN GmbH. All Rights Reserved.

File	panax HRM_ITS2(2).rex
Operator	
Run Id	
Notes	
Machine Serial No	814137
Channel	Gain
Green	5
Yellow	5
Orange	5
Red	5
HRM	-2
Crimson	7
Channel	Threshold
Cycling A.Green (Page 1)	
HRM A.HRM (Page	0

Melt analysis of HRM A.HRM (Page 1)

No.	Color	Name	Genotype	Peak 1	Peak 2	Peak 3	Peak 4	Peak 5	Peak 6	Peak 7
1	255	QBG Phytolacca americana		60.65	61.13	61.53	62.05	62.35	62.97	63.73
2	51400	QBG Phytolacca japonica		60.5	60.92	61.45	62.28	62.88	63.32	63.85
3	16711680	QBG Talinum crassifolium		60.53	61.17	61.75	62.12	62.85	63.95	64.85
4	8388736	QBG Talinum fruticosum		60.83	61.33	61.75	62.22	62.95	63.47	64.02
5	16744703	QBG Talinum paniculatum		60.42	61.1	61.37	62.25	63.05	63.42	63.97
6	16744448	QBG Talinum triangulare		60.45	61	61.27	61.72	62.58	63.05	63.43
7	8421376	Other		61	61.3	61.73	62.12	62.85	63.35	63.85
8	8421631	Other		60.55	61.03	61.5	62.08	63.05	63.82	64.35
9	1677088	Other		60.52	61.35	61.75	62.15	62.58	63.03	63.38
10	16711935	Other		60.4	61.35	62.25	62.6	63.15	63.45	64.23

11	197379	Other	60.5	61.05	61.23	61.63	62.13	62.67	63.05
12	13158400	Other	60.5	60.95	61.48	61.83	62.28	62.88	63.35
13	8504538	Other	60.88	61.3	62.1	62.32	62.95	63.87	64.65
14	8510085	Other	60.9	61.5	62.15	62.45	63.07	63.55	64.3
15	13491072	Panax ginseng root dry	60.35	61.1	61.67	62.2	62.55	63.05	63.48
16	14395776	Other	60.92	61.3	61.65	62.28	63	63.47	63.87
17	14450322	Panax notoginseng	60.45	60.9	61.35	62.02	62.85	63.4	63.8
No.	Color	Name	Peak 8	Peak 9	Peak 10	Peak 11	Peak 12	Peak 13	Peak 14
1	255	QBG Phytolacca americana	64.08	64.68	64.9	65.33	66.15	66.98	67.38
2	51400	QBG Phytolacca japonica	65.1	65.8	66.25	66.65	67.2	67.85	68.53
3	16711680	QBG Talinum crassifolium	65.23	65.75	66.28	66.4	67.03	67.45	68.15
4	8388736	QBG Talinum fruticosum	64.38	64.77	65.07	65.52	65.97	66.53	66.83
5	16744703	QBG Talinum paniculatum	64.33	64.8	65.37	66.05	66.95	67.65	67.92
6	16744448	QBG Talinum triangulare	63.92	64.25	64.73	65.18	65.67	66.2	66.73
7	8421376	Other	64.43	64.9	65.3	65.8	66.18	66.6	67.23
8	8421631	Other	64.8	65.22	65.77	66.18	66.45	67.05	67.32
9	1677088	Other	63.88	64.32	64.8	65.25	65.62	66.03	66.8
10	16711935	Other	64.82	65.32	65.97	66.35	66.88	67.8	68.35
11	197379	Other	63.37	63.78	64.2	64.75	65.33	65.9	66.3
12	13158400	Other	63.87	64.22	64.55	64.92	65.3	65.82	66.38
13	8504538	Other	65.1	66.1	66.52	67	67.4	68.3	68.9
14	8510085	Other	64.75	65.07	65.53	65.97	66.87	67.58	67.92
15	13491072	Panax ginseng root dry	64.15	64.75	65.28	66	66.4	66.93	67.48
16	14395776	Other	64.15	64.7	65.28	65.83	66.27	66.7	67.13
17	14450322	Panax notoginseng	64.07	64.57	65.22	65.75	66.45	66.92	67.25
No.	Color	Name	Peak 15	Peak 16	Peak 17	Peak 18	Peak 19	Peak 20	Peak 21
1	255	QBG Phytolacca americana	67.75	68.23	68.65	69.35	69.77	70.6	71.27
2	51400	QBG Phytolacca japonica	69.02	69.85	70.4	70.8	71.2	71.6	72.2
3	16711680	QBG Talinum crassifolium	68.43	68.82	69.3	69.9	70.7	71.18	71.7
4	8388736	QBG Talinum fruticosum	67.47	67.93	68.35	69.02	69.43	70.03	70.7
5	16744703	QBG Talinum paniculatum	68.38	68.6	69.7	70.07	70.48	71.03	71.72
6	16744448	QBG Talinum triangulare	67.28	67.78	68.53	69.18	69.65	70.17	70.7
7	8421376	Other	67.73	68.2	68.45	69.18	69.88	70.22	70.8
8	8421631	Other	67.8	68.12	68.55	69.28	69.82	70.42	70.83
9	1677088	Other	67.45	67.88	68.3	69.32	69.75	70.12	70.7
10	16711935	Other	68.7	69.2	69.73	70.43	71	71.5	72

11	197379	Other	66.67	67.25	67.83	68.45	69.18	69.7	70.15
12	13158400	Other	66.93	67.28	67.92	68.43	68.8	69.28	69.97
13	8504538	Other	69.88	70.63	71.18	71.65	72.43	72.9	73.15
14	8510085	Other	68.4	68.88	69.35	69.8	70.48	71.07	71.45
15	13491072	Panax ginseng root dry	67.83	68.55	69.1	69.78	70.18	70.95	71.38
16	14395776	Other	67.75	68.62	69.23	69.7	70.23	70.78	71.25
17	14450322	Panax notoginseng	67.72	68.1	68.4	68.8	69.27	69.93	70.32

No.	Color	Name	Peak 22	Peak 23	Peak 24	Peak 25	Peak 26	Peak 27	Peak 28
1	255	QBG Phytolacca americana	71.92	72.42	72.88	73.6	74.02	74.45	74.9
2	51400	QBG Phytolacca japonica	72.95	73.58	74.02	74.35	74.95	75.53	76.08
3	16711680	QBG Talinum crassifolium	72.38	72.85	73.5	73.92	74.4	74.88	75.33
4	8388736	QBG Talinum fruticosum	70.83	71.72	72.4	72.93	73.7	74.17	74.57
5	16744703	QBG Talinum paniculatum	72.07	72.62	73.43	73.9	74.7	75.1	75.65
6	16744448	QBG Talinum triangulare	71.08	71.25	72.17	72.73	73.05	73.67	74.05
7	8421376	Other	71.25	71.65	72.13	72.9	73.25	73.58	74.03
8	8421631	Other	71.2	71.63	71.87	72.38	72.88	73.47	74.03
9	1677088	Other	71.23	71.7	72.12	72.62	73.03	73.72	74.35
10	16711935	Other	72.65	73.18	74.17	74.6	75.15	75.65	76.35
11	197379	Other	71.22	71.82	72.22	72.8	72.95	73.75	74.15
12	13158400	Other	70.25	71.22	71.68	71.95	72.33	72.85	73.57
13	8504538	Other	73.32	73.6	74.1	74.87	75.35	75.83	76.6
14	8510085	Other	72.2	72.63	72.97	73.88	74.68	75.25	75.82
15	13491072	Panax ginseng root dry	72.05	72.45	72.9	73.28	73.48	73.95	74.12
16	14395776	Other	71.72	72.2	72.82	73.17	73.47	74.18	74.63
17	14450322	Panax notoginseng	70.83	71.28	71.57	72.07	72.8	73.35	73.5

No.	Color	Name	Peak 29	Peak 30	Peak 31	Peak 32	Peak 33	Peak 34	Peak 35
1	255	QBG Phytolacca americana	75.68	76.8	77.5	78.22	78.92	80.08	80.67
2	51400	QBG Phytolacca japonica	76.52	76.9	77.4	77.75	78.13	78.55	79.6
3	16711680	QBG Talinum crassifolium	75.78	76.1	76.55	77	77.4	77.87	78.2
4	8388736	QBG Talinum fruticosum	75.05	75.77	76.45	77.13	77.55	78	78.28
5	16744703	QBG Talinum paniculatum	76.35	76.75	77.25	77.65	78.1	78.98	79.6
6	16744448	QBG Talinum triangulare	74.67	75.07	75.6	76.15	76.53	76.87	77.3
7	8421376	Other	74.48	74.95	75.57	75.78	76.23	76.92	77.47
8	8421631	Other	74.33	74.92	75.35	75.83	76.62	77.07	77.48
9	1677088	Other	74.78	75.13	75.75	76.55	77.13	77.6	78.02
10	16711935	Other	76.85	77.2	77.7	78.32	78.95	79.5	80.07

11	197379	Other	74.58	75	75.58	76.18	76.87	77.35	77.97
12	13158400	Other	73.93	74.37	74.9	75.43	76.1	76.52	76.95
13	8504538	Other	77.07	77.47	77.85	78.27	78.95	79.35	79.9
14	8510085	Other	76.5	77.17	78	78.32	78.78	79.08	79.73
15	13491072	Panax ginseng root dry	74.65	75.15	75.65	76.33	76.78	77.23	77.73
16	14395776	Other	75	75.52	76.27	76.9	77.3	77.8	78.07
17	14450322	Panax notoginseng	74.03	74.88	75.45	75.88	76.5	76.98	77.47

No.	Color	Name	Peak 36	Peak 37	Peak 38	Peak 39	Peak 40	Peak 41	Peak 42
1	255	QBG Phytolacca americana	81.2	81.82	82.37	82.83	83.5	83.9	84.6
2	51400	QBG Phytolacca japonica	80.2	80.8	81.12	81.55	82.08	83.2	84.75
3	16711680	QBG Talinum crassifolium	78.63	79.3	79.83	80.18	80.78	81.05	81.45
4	8388736	QBG Talinum fruticosum	79.05	79.72	80.03	80.55	81	81.58	81.9
5	16744703	QBG Talinum paniculatum	80.1	80.5	80.95	81.25	81.73	82.33	82.8
6	16744448	QBG Talinum triangulare	77.77	78.12	78.68	79.5	80.15	80.85	81.42
7	8421376	Other	78.15	78.53	78.85	79.28	79.92	80.35	80.87
8	8421631	Other	77.85	78.25	78.73	79.35	79.77	80.18	80.7
9	1677088	Other	78.32	79.25	79.8	80.65	81.55	82.03	82.48
10	16711935	Other	80.53	80.9	81.3	81.83	82.37	82.8	83.42
11	197379	Other	78.65	79.5	80.13	80.75	81.23	82.15	82.87
12	13158400	Other	77.45	77.8	78.2	78.55	79.42	79.87	80.32
13	8504538	Other	80.12	80.6	81.15	82.15	82.95	83.67	84.35
14	8510085	Other	80.05	80.55	81.08	81.3	82.02	82.38	82.78
15	13491072	Panax ginseng root dry	78.07	78.9	79.52	80.07	80.55	80.93	81.25
16	14395776	Other	78.6	79.18	79.53	80.28	81.15	81.58	82.13
17	14450322	Panax notoginseng	77.83	78.25	78.88	79.35	79.85	80.3	81.1

No.	Color	Name	Peak 43	Peak 44	Peak 45	Peak 46	Peak 47	Peak 48	Peak 49
1	255	QBG Phytolacca americana	85.55	87.2	89	89.55			
2	51400	QBG Phytolacca japonica	85.35	86.8	87.28	88.35	89.35		
3	16711680	QBG Talinum crassifolium	81.85	82.55	83.05	83.8	84.55	85.13	85.93
4	8388736	QBG Talinum fruticosum	82.37	82.78	83.45	84.1	84.67	85.38	85.85
5	16744703	QBG Talinum paniculatum	83.48	85.17	86.97	87.57	88.23	89.4	
6	16744448	QBG Talinum triangulare	82.15	82.87	83.48	84.5	84.97	86.7	87.35
7	8421376	Other	82.08	83.02	83.22	83.72	84.4	85.15	85.63
8	8421631	Other	81.13	81.57	82.8	83.8	84.13	84.57	85.87
9	1677088	Other	82.92	83.47	83.85	84.42	84.92	85.4	85.88
10	16711935	Other	85.05	85.55	85.92	87.05	87.42	87.67	88.17

11	197379	Other	83.5	84.02	84.43	85.15	85.68	86.27	87.4
12	13158400	Other	80.77	81.15	81.95	82.18	82.87	83.57	83.98
13	8504538	Other	84.67	85.03	85.45	85.82	86.3	86.52	86.82
14	8510085	Other	84.75	85.38	85.87	87.6	88.2	88.55	89
15	13491072	Panax ginseng root dry	81.78	82.35	82.88	83.42	85.15	85.87	87.38
16	14395776	Other	82.55	82.97	83.57	84.45	84.85	87.33	
17	14450322	Panax notoginseng	81.5	81.98	82.1	82.68	83.13	83.65	84.15

No.	Color	Name	Peak 50	Peak 51	Peak 52	Peak 53	Peak 54	Peak 55	Peak 56
1	255	QBG Phytolacca americana							
2	51400	QBG Phytolacca japonica							
3	16711680	QBG Talinum crassifolium	86.45	87.2	87.65				
4	8388736	QBG Talinum fruticosum	86.72	87.15	87.63	88.23	89.05	89.42	
5	16744703	QBG Talinum paniculatum							
6	16744448	QBG Talinum triangulare	89.08						
7	8421376	Other	86	86.55	87.25	87.97	88.4	88.82	89.35
8	8421631	Other	86.22	86.8	87.42	87.98	89.25	89.85	
9	1677088	Other	86.22	86.78	87.17	87.62	88.15	89.1	89.63
10	16711935	Other	88.5	89.02	89.57				
11	197379	Other	88	89.42	89.8				
12	13158400	Other	84.85	85.23	86.55	87.25	88.4	88.75	89.3
13	8504538	Other	87.3	87.82	88.38	89.1	89.57		
14	8510085	Other	89.32						
15	13491072	Panax ginseng root dry	88.13	88.85	89.5				
16	14395776	Other							
17	14450322	Panax notoginseng	85.15	86.1	86.98	87.25	87.9	89.27	

No.	Color	Name	Peak 57
1	255	QBG Phytolacca americana	
2	51400	QBG Phytolacca japonica	
3	16711680	QBG Talinum crassifolium	
4	8388736	QBG Talinum fruticosum	
5	16744703	QBG Talinum paniculatum	
6	16744448	QBG Talinum triangulare	
7	8421376	Other	89.75
8	8421631	Other	
9	1677088	Other	
10	16711935	Other	

11	197379	Other	
12	13158400	Other	89.75
13	8504538	Other	
14	8510085	Other	
15	13491072	Panax ginseng root dry	
16	14395776	Other	
17	14450322	Panax notoginseng	

ITS(2)

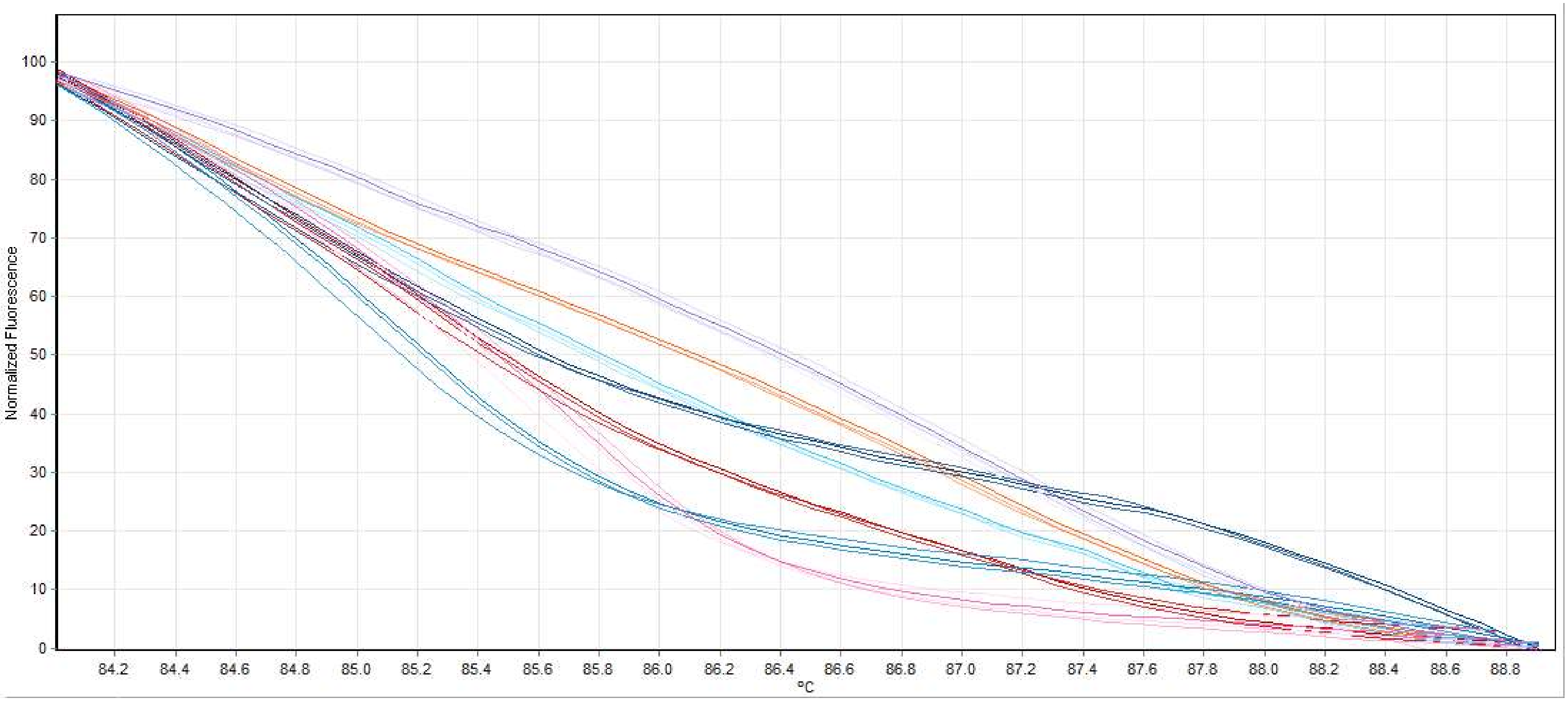

Excel Analysed Data Export
Copyright (c) 2013 QIAGEN GmbH. All Rights Reserved.

File	panax HRM_ITS2(3).rex

Operator	
Run Id	
Notes	
Machine Serial No	814137

Channel	Gain
Green	5
Yellow	5
Orange	5
Red	5
HRM	-2.33333
Crimson	7

Channel	Threshold
Cycling A.Green (Page 1)	
HRM A.HRM (Page	0

Melt analysis of HRM A.HRM (Page 1)

No.	Color	Name	Genotype	Peak 1	Peak 2	Peak 3	Peak 4	Peak 5	Peak 6	Peak 7
1	255	QBG Phytolacca americana		60.2	60.68	61.03	61.52	62.32	62.68	62.97
2	51400	QBG Phytolacca japonica		60.77	61.18	61.9	62.9	63.55	64.22	64.7
3	16711680	QBG Talinum crassifolium		60.35	61.08	61.52	62.28	63.08	63.68	64.1
4	8388736	QBG Talinum fruticosum		60.2	60.7	61.25	61.53	61.98	62.45	63.1
5	16744703	QBG Talinum paniculatum		60.55	60.95	61.57	62.35	62.9	63.55	63.92
6	16744448	QBG Talinum triangulare		60.88	61.32	61.55	62.5	62.88	63.4	64.12
7	8421376	Talinum crassifolium		60.98	61.43	62.08	62.6	63.03	63.47	64.22
8	8421631	Other		60.25	60.75	61.23	61.55	62.15	62.53	63.1
9	1677088	Other		60.3	61.35	62.18	63.02	63.92	64.45	65.03
10	16711935	Other		60.55	61.3	61.45	61.65	62.12	62.9	63.72
11	197379	Panax ginseng		60.85	61.55	62.18	62.87	63.4	63.65	64.7
12	13158400	Panax notoginseng		60.3	61.23	61.77	62.33	62.87	63.48	64.15
13	8504538	Talinum crassifolium		60.35	61.22	62.22	62.47	62.6	63.18	63.75

No.	Color	Name	Genotype							
14	8510085	dH2O		60.25	60.52	61.52	62.25	63.05	63.85	64.43

No.	Color	Name	Genotype	Peak 8	Peak 9	Peak 10	Peak 11	Peak 12	Peak 13	Peak 14
1	255	QBG Phytolacca americana		63.35	63.92	64.45	65.17	65.7	66.08	66.82
2	51400	QBG Phytolacca japonica		65.27	65.8	66.6	67.37	67.9	69.12	69.65
3	16711680	QBG Talinum crassifolium		64.65	65.15	65.93	66.72	67.07	67.6	67.95
4	8388736	QBG Talinum fruticosum		64	64.47	64.75	65.2	65.73	66.45	67.02
5	16744703	QBG Talinum paniculatum		64.43	64.92	65.32	65.7	66.52	67.2	67.52
6	16744448	QBG Talinum triangulare		64.8	65.5	65.98	66.47	66.82	67.4	67.9
7	8421376	Talinum crassifolium		64.75	65.3	65.93	66.45	67.12	67.45	67.9
8	8421631	Other		63.57	64.05	64.77	65.18	65.8	66.15	66.67
9	1677088	Other		65.75	66.65	67.15	67.62	68	68.57	69.37
10	16711935	Other		64.2	64.47	65.4	65.98	66.52	67.25	67.9
11	197379	Panax ginseng		65.27	66	66.55	67.17	67.4	67.85	68.23
12	13158400	Panax notoginseng		64.67	64.85	65.23	65.9	66.42	67.15	67.53
13	8504538	Talinum crassifolium		64.12	64.75	65.17	65.95	66.57	66.95	67.72
14	8510085	dH2O		64.98	65.52	66.47	67.1	67.62	68.53	68.88

No.	Color	Name	Genotype	Peak 15	Peak 16	Peak 17	Peak 18	Peak 19	Peak 20	Peak 21
1	255	QBG Phytolacca americana		67.27	67.8	68.75	69.25	70	70.73	71.1
2	51400	QBG Phytolacca japonica		70.15	70.75	71.3	71.75	72.2	72.78	73.15
3	16711680	QBG Talinum crassifolium		68.4	68.9	69	69.7	70.17	70.98	71.37
4	8388736	QBG Talinum fruticosum		67.67	68.35	68.7	69.35	69.88	70.4	70.9
5	16744703	QBG Talinum paniculatum		67.98	68.48	69	69.45	69.95	70.4	70.85
6	16744448	QBG Talinum triangulare		68.23	68.63	69.1	69.55	70.28	70.75	71.25
7	8421376	Talinum crassifolium		68.25	68.67	69.1	69.7	70.22	70.63	71.32
8	8421631	Other		67.13	67.8	68.43	69.15	69.7	70.37	70.95
9	1677088	Other		69.85	70.45	70.97	71.57	72.45	72.75	73.15
10	16711935	Other		68.2	68.75	69.47	69.97	70.43	70.83	71.23
11	197379	Panax ginseng		68.75	69.1	69.55	70.17	70.65	71.22	71.87
12	13158400	Panax notoginseng		67.88	68.27	68.62	69.12	69.72	70.17	70.85
13	8504538	Talinum crassifolium		68.47	68.77	69.25	69.67	70.1	70.45	70.65
14	8510085	dH2O		69.47	69.93	70.4	70.97	71.6	72.5	73

No.	Color	Name	Genotype	Peak 22	Peak 23	Peak 24	Peak 25	Peak 26	Peak 27	Peak 28
1	255	QBG Phytolacca americana		71.77	72.25	72.67	73.12	73.77	74.75	75.35
2	51400	QBG Phytolacca japonica		73.9	74.73	75.03	75.35	75.53	76.35	76.75
3	16711680	QBG Talinum crassifolium		72.32	72.88	73.18	73.6	74.22	74.73	75.32
4	8388736	QBG Talinum fruticosum		71.5	72.22	72.63	73	73.62	74.27	74.73

5	16744703	QBG Talinum paniculatum		71.13	71.77	72.13	72.57	72.85	73.48	73.95
6	16744448	QBG Talinum triangulare		71.8	72.25	72.8	73.47	73.97	75	75.68
7	8421376	Talinum crassifolium		72.18	72.85	73.3	73.77	74	74.77	75.15
8	8421631	Other		71.4	72.3	72.75	73.18	73.68	74.2	74.62
9	1677088	Other		73.55	74.25	74.8	75.35	75.98	76.43	76.9
10	16711935	Other		71.78	72.28	72.52	73.05	73.43	73.9	74.55
11	197379	Panax ginseng		72.85	73.35	73.9	74.6	74.98	75.43	76.05
12	13158400	Panax notoginseng		71.3	72.18	72.83	73.25	73.72	74.03	74.75
13	8504538	Talinum crassifolium		71.5	72.27	72.72	73.65	74.27	74.65	75.38
14	8510085	dH2O		73.55	73.98	74.55	74.83	75.45	75.85	76.92

No.	Color	Name	Genotype	Peak 29	Peak 30	Peak 31	Peak 32	Peak 33	Peak 34	Peak 35
1	255	QBG Phytolacca americana		75.8	76.42	76.95	77.4	77.88	78.55	78.87
2	51400	QBG Phytolacca japonica		77.2	77.8	78.35	78.85	79.5	80.03	80.35
3	16711680	QBG Talinum crassifolium		75.65	76.45	77.08	77.4	77.85	78.67	79.15
4	8388736	QBG Talinum fruticosum		75.32	75.88	76.4	76.8	77.22	77.75	78.58
5	16744703	QBG Talinum paniculatum		74.55	74.97	75.55	75.73	76.22	76.93	77.55
6	16744448	QBG Talinum triangulare		75.98	76.45	76.85	77.37	78.18	78.6	79.05
7	8421376	Talinum crassifolium		75.68	75.92	76.42	77.25	77.87	78.4	79.03
8	8421631	Other		75.62	76.68	77.12	77.6	78.5	79.45	79.87
9	1677088	Other		77.6	78.05	78.55	78.92	79.42	80.45	80.95
10	16711935	Other		75.05	75.7	76.15	76.9	77.25	77.6	78.05
11	197379	Panax ginseng		76.45	76.85	77.35	77.72	78.18	78.57	79.13
12	13158400	Panax notoginseng		75.23	75.65	76.32	76.65	77.25	77.87	78.47
13	8504538	Talinum crassifolium		75.63	76.5	77.02	77.5	77.9	78.45	79.25
14	8510085	dH2O		77.72	78.1	78.67	78.9	79.33	80	80.9

No.	Color	Name	Genotype	Peak 36	Peak 37	Peak 38	Peak 39	Peak 40	Peak 41	Peak 42
1	255	QBG Phytolacca americana		79.42	80.2	80.82	81.27	82.1	82.85	83.33
2	51400	QBG Phytolacca japonica		80.8	81.3	81.67	82.18	83.05	83.6	84.03
3	16711680	QBG Talinum crassifolium		79.78	80.6	81.25	82.1	82.7	83.13	84.3
4	8388736	QBG Talinum fruticosum		79.25	79.95	80.5	80.95	81.53	82.08	82.37
5	16744703	QBG Talinum paniculatum		78.05	78.67	80.03	80.35	80.87	81.5	82.38
6	16744448	QBG Talinum triangulare		79.45	79.85	80.35	80.85	81.62	82.35	82.95
7	8421376	Talinum crassifolium		79.42	79.8	80.3	80.78	81.05	81.82	82.65
8	8421631	Other		80.37	80.63	81.15	82.17	82.53	84.25	84.77
9	1677088	Other		81.47	82.12	82.38	82.83	83.55	84.18	84.65
10	16711935	Other		78.55	78.93	79.5	80.07	80.45	80.82	81.37

11	197379	Panax ginseng		79.63	80.33	80.85	81.78	82.43	83.23	83.75
12	13158400	Panax notoginseng		79.05	79.85	80.35	80.63	81.05	81.88	82.35
13	8504538	Talinum crassifolium		79.93	80.65	80.97	81.25	82.1	82.83	83.48
14	8510085	dH2O		81.5	82.32	82.8	83.3	83.83	84.25	84.62

No.	Color	Name	Genotype	Peak 43	Peak 44	Peak 45	Peak 46	Peak 47	Peak 48	Peak 49
1	255	QBG Phytolacca americana		83.75	84.17	84.55	84.9	85.98	86.73	87.15
2	51400	QBG Phytolacca japonica		84.45	84.93	85.57	86.32	86.7	87	88.95
3	16711680	QBG Talinum crassifolium		84.82	85.83	86.48	86.82	87.2	87.65	89.07
4	8388736	QBG Talinum fruticosum		82.8	83.5	84.3	84.75	85.78	86.28	86.92
5	16744703	QBG Talinum paniculatum		82.92	83.35	84.03	84.72	85.75	86.03	86.75
6	16744448	QBG Talinum triangulare		83.25	84.35	84.9	85.25	85.65	86.18	86.65
7	8421376	Talinum crassifolium		83.48	84.05	84.72	85.6	86.58	87.03	87.33
8	8421631	Other		85.9	86.48	86.82	87.15	87.57	89.1	89.55
9	1677088	Other		85.08	85.55	86.4	86.92	87.42	88.67	89.3
10	16711935	Other		81.83	82.42	82.85	83.3	84.07	84.45	84.8
11	197379	Panax ginseng		84.32	84.92	86.13	86.63	87.07	87.77	89.3
12	13158400	Panax notoginseng		82.6	83.07	83.48	84	84.45	84.97	85.62
13	8504538	Talinum crassifolium		83.88	84.25	84.8	85.12	85.8	86.5	86.95
14	8510085	dH2O		85.17	85.98	86.3	86.8	87.5	88.3	88.7

No.	Color	Name	Genotype	Peak 50	Peak 51	Peak 52	Peak 53	Peak 54	Peak 55	Peak 56
1	255	QBG Phytolacca americana		87.65	88.15	88.75	89.6	89.95	91.65	
2	51400	QBG Phytolacca japonica		89.55	90.15	90.78	91.18	91.63		
3	16711680	QBG Talinum crassifolium		89.62	89.92	90.48	91.02	91.52		
4	8388736	QBG Talinum fruticosum		88.02	88.72	89.25	89.83	90.6	91.02	91.4
5	16744703	QBG Talinum paniculatum		87.62	88.05	88.75	89.3	89.8	90.07	90.85
6	16744448	QBG Talinum triangulare		87.08	87.45	89.25	90.35	90.8	91.18	
7	8421376	Talinum crassifolium		89.03	89.55	90.12	90.95	91.55		
8	8421631	Other		89.92	90.45	91.05	91.53			
9	1677088	Other		89.82	90.58	91.15	91.4			
10	16711935	Other		85.27	86.15	86.75	88.82	89.3	90.63	91.4
11	197379	Panax ginseng		90.05	90.25	90.73	91.22	91.8		
12	13158400	Panax notoginseng		86.98	87.4	88.35	88.93	89.57	90.13	90.88
13	8504538	Talinum crassifolium		87.17	87.62	88.6	89.08	90.47	91.55	
14	8510085	dH2O		89.78	90.55	91.42				

No.	Color	Name	Genotype	Peak 57	Peak 58
1	255	QBG Phytolacca americana			

2	51400	QBG Phytolacca japonica		
3	16711680	QBG Talinum crassifolium		
4	8388736	QBG Talinum fruticosum		
5	16744703	QBG Talinum paniculatum	91.3	
6	16744448	QBG Talinum triangulare		
7	8421376	Talinum crassifolium		
8	8421631	Other		
9	1677088	Other		
10	16711935	Other		
11	197379	Panax ginseng		
12	13158400	Panax notoginseng	91.18	91.55
13	8504538	Talinum crassifolium		
14	8510085	dH2O		

ITS(3)

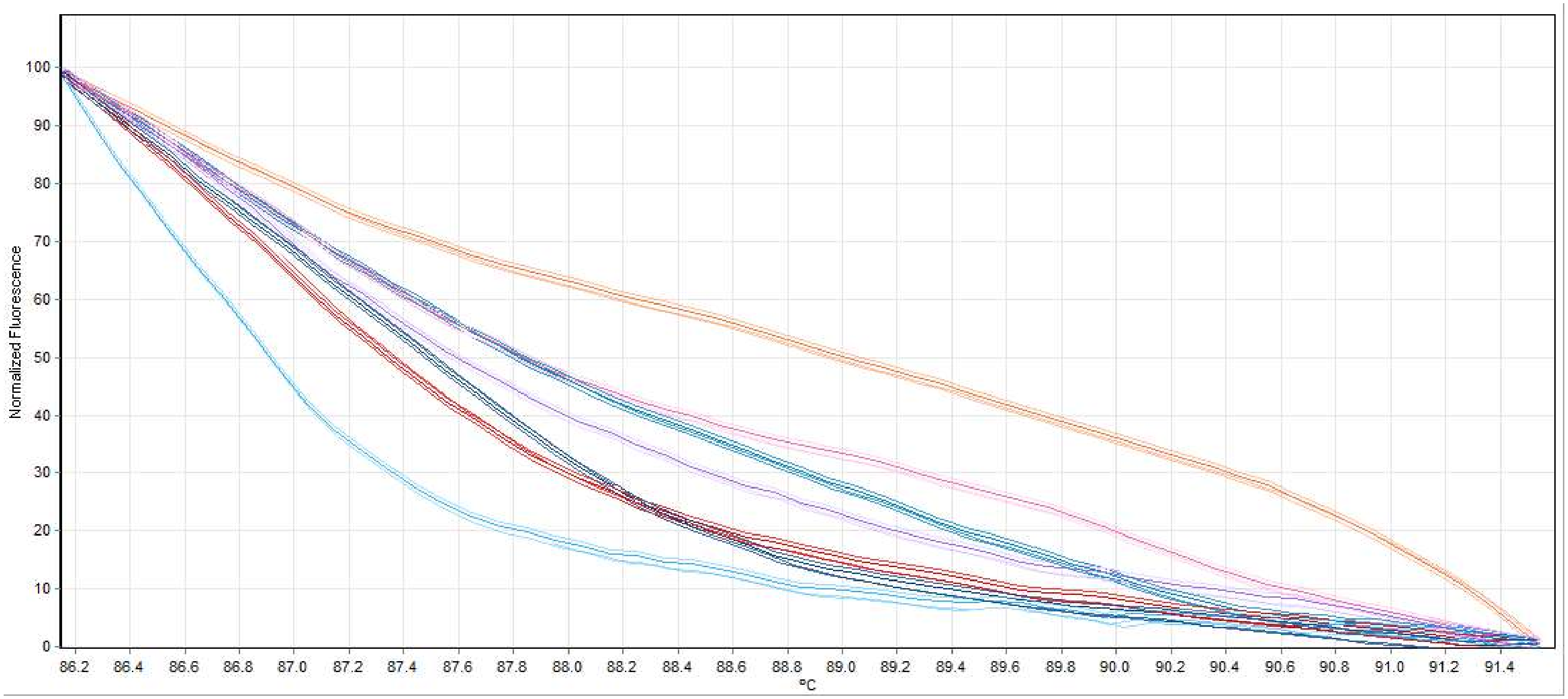

Supplement: File S1 [file peerj-07-7660-s001.pdf]
